# Self-assembling peptide hydrogels functionalized with LN- and BDNF- mimicking epitopes synergistically enhance peripheral nerve regeneration

**DOI:** 10.7150/thno.44276

**Published:** 2020-07-09

**Authors:** Shuhui Yang, Chong Wang, Jinjin Zhu, Changfeng Lu, Haitao Li, Fuyu Chen, Jiaju Lu, Zhe Zhang, Xiaoqing Yan, He Zhao, Xiaodan Sun, Lingyun Zhao, Jing Liang, Yu Wang, Jiang Peng, Xiumei Wang

**Affiliations:** 1 State Key Laboratory of New Ceramics and Fine Processing, Key Laboratory of Advanced Materials of Ministry of Education, School of Materials Science and Engineering, Tsinghua University, Beijing 100084, China.; 2 Institute of Orthopedics, Chinese PLA General Hospital, Beijing 100853, China; Co-innovation Center of Neuroregeneration, Nantong University, Nantong, Jiangsu Province 226007, China.; 3 Department of Orthopaedic Surgery, Sir Run Run Shaw Hospital, Zhejiang University School of Medicine & Key Laboratory of Musculoskeletal System Degeneration and Regeneration Translational Research of Zhejiang, Hangzhou 310016, China.; 4 Department of Orthopaedics and Trauma, Peking University People's Hospital, Beijing 100191, China.; 5 School of Clinical Medicine, Tsinghua University, Beijing 100084, China.; 6 Department of Pediatrics, Tianjin Hospital, Tianjin University, No. 406 Jiefang Nan Road, Tianjin 300211, China.

**Keywords:** peripheral nerve regeneration, self-assembling peptide, neurotrophic peptide, laminin, nanofiber hydrogel

## Abstract

The regenerative capacity of the peripheral nervous system is closely related to the role that Schwann cells (SCs) play in construction of the basement membrane containing multiple extracellular matrix proteins and secretion of neurotrophic factors, including laminin (LN) and brain-derived neurotrophic factor (BDNF). Here, we developed a self-assembling peptide (SAP) nanofiber hydrogel based on self-assembling backbone Ac-(RADA)_4_-NH_2_ (RAD) dual-functionalized with laminin-derived motif IKVAV (IKV) and a BDNF-mimetic peptide epitope RGIDKRHWNSQ (RGI) for peripheral nerve regeneration, with the hydrogel providing a three-dimensional (3D) microenvironment for SCs and neurites.

**Methods:** Circular dichroism (CD), atomic force microscopy (AFM), and scanning electron microscopy (SEM) were used to characterize the secondary structures, microscopic structures, and morphologies of self-assembling nanofiber hydrogels. Then the SC adhesion, myelination and neurotrophin secretion were evaluated on the hydrogels. Finally, the SAP hydrogels were injected into hollow chitosan tubes to bridge a 10-mm-long sciatic nerve defect in rats, and *in vivo* gene expression at 1 week, axonal regeneration, target muscular re-innervation, and functional recovery at 12 weeks were assessed.

**Results:** The bioactive peptide motifs were covalently linked to the C-terminal of the self-assembling peptide and the functionalized peptides could form well-defined nanofibrous hydrogels capable of providing a 3D microenvironment similar to native extracellular matrix. SCs displayed improved cell adhesion on hydrogels with both IKV and RGI, accompanied by increased cell spreading and elongation relative to other groups. RSCs cultured on hydrogels with IKV and RGI showed enhanced gene expression of NGF, BDNF, CNTF, PMP22 and NRP2, and decreased gene expression of NCAM compared with those cultured on other three groups after a 7-day incubation. Additionally, the secretion of NGF, BDNF, and CNTF of RSCs was significantly improved on dual-functionalized peptide hydrogels after 3 days. At 1 week after implantation, the expressions of neurotrophin and myelin-related genes in the nerve grafts in SAP and Autograft groups were higher than that in Hollow group, and the expression of S100 in groups containing both IKV and RGI was significantly higher than that in groups containing either IKV or RGI hydrogels, suggesting enhanced SC proliferation. The morphometric parameters of the regenerated nerves, their electrophysiological performance, the innervated muscle weight and remodeling of muscle fibers, and motor function showed that RAD/IKV/RGI and RAD/IKV-GG-RGI hydrogels could markedly improve axonal regeneration with enhanced re-myelination and motor functional recovery through the synergetic effect of IKV and RGI functional motifs.

**Conclusions:** We found that the dual-functionalized SAP hydrogels promoted RSC adhesion, myelination, and neurotrophin secretion *in vitro* and successfully bridged a 10-mm gap representing a sciatic nerve defect in rats *in vivo*. The results demonstrated the synergistic effect of IKVAV and RGI on axonal regrowth and function recovery after peripheral nerve injury.

## Introduction

Peripheral nerve injury (PNI) causes severe motor disability, sensory aberrations, and pain syndromes in patients, seriously restricting social development 1-3. Autologous nerve grafting is currently regarded as the gold standard for reconstruction of nerve damage in the clinic 4. However, autografts are limited by considerable disadvantages, including poor functional recovery, finite availability, donor site function loss, morbidity, and pain 5. Nerve guidance conduits (NGCs) have emerged as a potential alternative to autologous nerve grafts due to their ability to reduce neuroma formation and prevent axonal escape 6-8. Moreover, the repair of small defects is achievable using clinically available NGCs 9. However, effective and efficient functional reconstruction of larger and complex defects remains clinically challenging 10. Therefore, providing an effective method to bridge the gap in nerve regeneration represents a focus of research into peripheral nerve regeneration, especially the construction of a beneficial intraluminal microenvironment of NGCs 11.

In the pursuit of an effective nerve conduit, various physical and biological strategies have been employed 12. Progress in research associated with elucidating the intrinsic mechanism of peripheral nerve repair has increased the focus on the design of scaffold materials that simulate the repair process such as constructing the neurovascular microenvironment 13. Unlike axons in the central nervous system (CNS), injured peripheral nerves exhibit the capacity to regenerate largely due to the supportive population of Schwann cells (SCs), which are primary glial cells in the peripheral nervous system (PNS). SCs have a unique capacity to de-differentiate, re-enter the cell cycle, and subsequently myelinate regenerating axons, which can promote regeneration by secreting neurotrophic molecules and establishing a supportive growth matrix 14. SCs play an important role in peripheral nerve repair not only in the early clearance of debris and control of inflammation but also in axon growth and myelination 15. SCs play two main roles in the repair: clearing debris clearance and secretion of growth promoting matrix, neurotrophic factors, and cell adhesion molecules 16. On the one hand, the basement membrane constructed by SCs facilitates axon-to-axon and axon-to-SC attachment based in the presence of the extracellular matrix (ECM) proteins, such as laminin (LN) and fibronectin (FN), which represent a kind of neurite outgrowth promoting factors 17. On the other hand, matured myelinating SCs can also produce many neurotrophic factors, such as nerve growth factor (NGF), brain-derived neurotrophic factor (BDNF) and ciliary neurotrophic factor (CNTF), as well as their respective receptors. Both ECM proteins and neurotrophic factors are essential for axonal growth after injury: ECM proteins orient and provide an adhesive element for axons and SCs, whereas neurotrophic factors are the specific trophic agents that enhance peripheral nerve regeneration, with both of these sets of molecules required for regeneration 17, 18. Without either neurite outgrowth matrix or neurotrophic factors, regeneration will either not occur or be dysfunctional. This suggests a synergistic effect on nerve regeneration between growth promoting matrix and neurotrophin, which describes our design of the microenvironment within the conduits. Therefore, we hypothesize that modeling the microenvironment in the lumen of NGCs via the synergistic effects of neurotrophic and neurite outgrowth promoting factors is essential for peripheral nerve regeneration.

LN is a primary component of the basement membrane and heavily involved in nerve regeneration 19, 20. LN is secreted by SCs and acts as an adhesive component that promotes the regenerative capacity of basal lamina scaffolds following nerve injury, with previous studies demonstrating its ability to promote neuritogenesis *in vitro* and *in vivo* 21, 22. Several fragments of the LN sequence were recently identified and tested for their potential bioactivity, with IKVAV (Ile-Lys-Val-Ala-Val) shown to selectively promote SC migration *in vitro* and capable of easy conjugation to SAPs 23, 24. The functional peptide motif IKVAV is commonly used in neurotrophin releasing and stem cell loading during treatment of traumatic brain injury, spinal cord injury, and sciatic nerve injury due to its ability to enhance cell survival and adhesion, neurite outgrowth, and even angiogenesis 25-27. BDNF is a member of the neurotrophin family and enhances myelination in SCs, promotes neuronal survival and neurite outgrowth, and prevents neural death 28, 29. Studies indicate that BDNF promotes axonal regrowth after sciatic nerve injury and is closely related to motor recovery following PNI 30-33. Hassannejad et al. fabricated an IKVAV-functionalized peptide amphiphile (PA) hydrogel to release BDNF for spinal cord repair, resulting in considerable axon preservation and reduced astrogliosis at 6 weeks without any inflammatory response 26. However, the use of growth factors and neurotrophic factors is sometimes limited due to their high cost, short half-life, controversial sources, and vulnerability 34. Moreover, BDNF concentration directly influences axonal regeneration, with low exogenous BDNF doses enhancing motor neuron axon regeneration, whereas high levels inhibiting regeneration 35. To address this issue, we previously reported that a neurotrophic peptide sequence RGIDKRHWNSQ (RGI) derived from BDNF promoted rat sciatic nerve regeneration 36, 37. The BDNF-mimetic peptide motif designed based on solvent-exposed loops 3 and 4 of BDNF simulated neurite outgrowth and survival by binding to the TrkB and p75 neurotrophin receptor 38. The previous study proposed an SAP nanofiber hydrogel functionalized with BDNF mimicking peptide RGI to improve axon regeneration and motor functional recovery; however, its regenerative effect was not as good as that of autografting, indicating a limitation in the use of a single factor in repairing nerve injury 36. Other studies showed that combined use of RGI and other peptide motifs derived from growth factors, such as NGF and VEGF, promoted nerve regeneration 13, 37. Therefore, we expanded our previous efforts to establish synergetic scaffolds harboring LN and BDNF mimicking peptide motifs to simulate effects of the extracellular matrix and neurotrophic factors at the same time for evaluation in a rat model of sciatic nerve injury.

In this study, the purpose is to evaluate the effects of different functionalized self-assembling peptide hydrogels that modify the intraluminal environment of the nerve guidance conduit. It's better to choose an appropriate NGC that has been sufficiently evaluated. Chitosan-based nerve grafts have been widely used to repair peripheral nerve defects and achieved satisfactory clinical results due to its excellent performance in biocompatibility, permeability, plasticity and biodegradability 39-41. Additionally, the chitosan degradation products could facilitate peripheral nerve regeneration by improving macrophage-constructed microenvironments 42. The chitosan tube in this study has been verified to be helpful for peripheral nerve regeneration in previous studies 11, 13, 36, 37. Therefore, the chitosan tube was used as guidance conduit in this study. Self-assembling peptides (SAPs) can form hydrogels with a nanofibrous network structure mimicking ECM in certain physiological environments, thereby making them a promising option for lumen-filling materials 43-45. Ac-RADARADARADARADA-CONH_2_ (RAD) is a typical and widely used SAP based on its high degree of biocompatibility, low cytotoxicity, three-dimensional (3D) structure promoting cell growth, and good integration with different shapes of wounds. RAD hydrogel is a promising material to be used in controlled drug release, cell-based therapies, and tissue engineering 46, 47. RAD comprises repeated segments that include positively charged arginine (R), hydrophobic alanine (A), and negatively charged aspartic acid (D) 48, 49, and functional motifs such as IKVAV and RGI can be easily conjugated and extended to the C-terminal residue sequence of RAD to enrich its functionality 50. Herein, we constructed a novel scaffold comprising functionalized self-assembling peptides based on RAD with the LN-derived motif IKVAV and a BDNF-derived motif RGI for promoting peripheral nerve regeneration synergistically. We designed and synthesized the peptides RAD-IKV and RAD-RGI by directly extending the C-terminal of RAD via solid phase synthesis, followed by fabrication of the functionalized SAP scaffolds RAD/IKV and RAD/RGI via mixing RAD with RAD-IKV and RAD-RGI, respectively. Then we combined them at a ratio of 1:1 to form RAD/IKV/RGI. Additionally, we synthesized RAD-IKV-GG-RGI with both functional motifs covalently combined, and prepared a RAD/IKV-GG-RGI hydrogel for comparison. Circular dichroism (CD), atomic force microscopy (AFM), and scanning electron microscopy (SEM) were used to characterize the secondary structures, microscopic structures, and morphologies of self-assembling nanofiber hydrogels. Then the SC adhesion, myelin and neurotrophin secretion were evaluated on the hydrogels. Finally, the SAP hydrogels were injected into hollow chitosan tubes to bridge a 10-mm-long sciatic nerve defect in rats, and *in vivo* gene expression, axonal regeneration, target muscular re-innervation, and functional recovery were assessed at various time points.

## Methods

### Peptide synthesis and preparation of materials

The SAPs RAD (Ac-RADARADARADARADA-NH_2_), LN-mimicking peptide-modified SAP RAD-IKV (Ac-RADARADARADARADA-GG-IKVAV-NH_2_), BDNF-mimicking peptide-modified SAP RAD-RGI (Ac-RADARADARADARADA-GG-RGIDKRHWNSQ-NH_2_) and bifunctional mimicking peptides-modified SAP RAD-IKV-GG-RGI (Ac-RADARADARADARADA-GG-IKVAV-GG-RGIDKRHWNSQ-NH_2_) (purity > 90%) were custom-synthesized and purified by ChinaPeptides Co., Ltd (Shanghai, China) and Scilight-Peptide Co., Ltd (Beijing, China). Two glycine was added as a linker to increase the flexibility of the functional epitopes. The purity and identities of the peptides were confirmed by analytical high-performance liquid chromatography (HPLC) and electrospray ionization mass spectrometry (ESI-MS).

The peptide powders were dissolved in distilled water to the desired concentration of 1% (v/w), filter-sterilized with a syringe filter (0.22 μm HT Tuffrun membrane; Pall Crop., Ann Arbor, MI, USA), and sonicated for 30 min (VCX 130PB; Sonics, Newtown, CT, USA) for subsequent use. The peptide solutions used in this study were listed in **Table 1**. The functionalized peptide solutions of RAD/IKV, RAD/RGI, and RAD/IKV-GG-RGI were obtained by mixing 50% pure RAD solution with 50% RAD-IKV, RAD-RGI, and RAD-IKV-GG-RGI solutions, respectively. The solution of RAD/IKV/RGI was acquired by mixing 50% RAD/IKV solution and 50% RAD/RGI solution. The self-assembling peptide hydrogels were fabricated within cell-culture Transwell inserts (10-mm diameter; Millipore, Billerica, MA, USA), as described previously 37. Briefly, sterilized inserts were placed in a 24-well culture plate with 400 μL of Dulbecco's Modified Eagle Medium (DMEM, Gibco, Life Technologies, Carlsbad, CA, USA) in each well. After sufficient wetting of the polytetrafluoroethylene (PTEF) membrane on the bottom of the inserts, 100 μL of the peptide solution was added onto the inserts and incubated for 15 min at 37 °C to allow gelation. Another 400 μL of DMEM was then carefully added onto the hydrogel and incubated at 37 °C for 15 min. All medium in each well was changed at least four times every 15 minutes in order to equilibrate the hydrogel to physiological pH, followed by overnight incubation at 37 °C.

The chitosan conduits were prepared according to the patent by Peking University People's Hospital and Textile Science Institute of China (Patent No. 01136314.2).

### Circular dichroism analysis

The CD spectra of the peptides were measured using Chirascan plus (Applied Photophysics, Leatherhead, UK). Peptide solutions were diluted to working concentrations of 0.01% (w/v), and 200 μL of the diluted samples were added to a quartz cuvette with a 1-mm path length. CD spectra were recorded over the range 180 nm to 260 nm with a step size of 1 nm at room temperature. All samples were evaluated three times, and the data averaged. The secondary structure fractions were measured by CONTINLL algorithm in the software CDPro compared to a set of selected reference proteins (Ibasis3 [SP37], 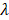 = 240 - 185 nm) 51.

### Atomic force microscopy analysis

Peptide solutions were diluted to a working solution of 0.01% (w/v), and the diluted samples were dropped onto a freshly cleaved mica surface and incubated for 30 s, followed by rinsing with 100 μL of distilled water. After air drying, the samples were immediately observed using an atomic force microscope (Bruker Dimension ICON; Bruker, Billerica, MA, USA) with a silicon scanning probe (OMCL-TR400PSA-1, triangular cantilever with reflect coating, length 200 μm, stiffness 0.02 N/m, resonant frequency 11 kHz; Olympus Corp., Tokyo, Japan) in contact mode. The scan area was with 2 μm × 2 μm with scan frequency of 1.00 Hz. The fiber widths of different peptides were measured by NanoScope Analysis 1.8 (Bruker Dimension ICON; Bruker, Billerica, MA, USA) 52.

### Scanning electron microscopy analysis

After gelation, the hydrogels were fixed with 2.5% glutaraldehyde for 2 h, washed twice in phosphate buffered saline (PBS), and dehydrated through successive ethanol washes with concentrations of 30%, 50%, 70%, 80%, 90%, 95%, and 100% (v/v) for 30 min in each bath. The samples were then dried using a CO_2_ critical point dryer (Samdri-PVT-3D; Tousimis, Rockville, MD, USA). The fresh fracture surfaces of the samples were sputter-coated with a layer of platinum in a sputter-coating chamber (EM ACE600; Leica, Wetzlar, Germany) and then imaged with a scanning electron microscope (Carl Zeiss, Oberkochen, Germany) at an accelerating voltage of 5 kV. To evaluate the pore size in the hydrogel, three random images in each group were selected. The pore sizes of different hydrogels were measured using Image Pro Plus 6.0 (Media Cybernetics, Silver Spring, MD, USA) according to a previous method 53.

### Rheological properties

Rheological properties of the hydrogels were measured using a Physica MCR301 rheometer (Anton Paar GmbH, Graz, Austria) using an 8-mm diameter parallel plate at room temperature (25 °C). The hydrogels in 1-mm height and 10-mm diameter were positioned on the sample loading stage, and the parallel plate was operated to contact with the hydrogel, followed with the scrape-off of the excess hydrogel beyond the plate. Stress/strain sweeps (0.01% - 100% at 1 Hz) were performed to identify the limits of the linear viscoelastic region of the hydrogels. Afterwards, in the linear viscoelastic region, a dynamic frequency sweep test (0.1 - 10 rad/s at 0.5% strain) was performed with a 0.75-mm truncation gap for each sample at room temperature. Each experiment was performed in triplicate.

### Schwann cell adhesion and morphology

Rat SCs (RSCs) were obtained from the National Infrastructure of Cell Line Resource (RSC-96; 3111C0001CCC000664; Beijing, China) and cultured in DMEM supplemented with 10% fetal bovine serum (FBS, Gibco, Life Technologies, Carlsbad, CA, USA) and 1% Penicillin-Streptomycin (PS, Gibco, Life Technologies, Carlsbad, CA, USA) (growth medium). RSCs were seeded on the surface of the hydrogels at a density of 2 × 10^4^ cells/well and the growth medium was changed every two days. After 7 days, RSCs were fixed with 4% paraformaldehyde for 30 min, permeated with 0.1% Triton X-100 solution for 5 min, and incubated in 1% bovine serum albumin (BSA, Cat No. A7030; Sigma-Aldrich, St. Louis, Mo, USA) for 30 min at 37 °C. Actin filaments and nuclei were stained with rhodamine-phalloidin (1:300; Cat No. PHDR1; Cytoskeleton, Denver, CO, USA) and 5 μM SYTOX green nucleic acid stain (Molecular Probes, Eugene, OR, USA), respectively. The cells were imaged using a confocal laser scanning microscope (LSM 710; Carl Zeiss). To evaluate cell adhesion and myelination on the hydrogels, three random images of each sample were selected, and the proportion of spreading cells (the number of cells in polarized and elongated morphology to the total number of cells) and cell elongation length were measured using Image Pro Plus 6.0 (Media Cybernetics). The cell elongation length was defined as the longest axis of the cell 54, 55.

### Quantitative real-time polymerase chain reaction (qRT-PCR)

Peptide solution (200 μL) was loaded directly into the wells of a 24-well plate, followed by the addition of 400 μL of culture medium onto the peptide solution and incubation for 15 min at 37 °C. After gelation, the medium was removed and changed at least twice in order to equilibrate the hydrogel to physiological pH. RSCs were seeded on the surface of the hydrogel at a density of 5 × 10^4^ cells/well (n = 3), and the medium was substituted with fresh medium every 2 days. After 7 days, total RNA from each sample was isolated using mRNA kit (DP501; Tiangen, Beijing, China) and then 500 ng of total RNA was reverse transcribed to cDNA using a FastQuant RT kit (KR-106; Tiangen). qPCR was performed using iTaq SYBR Green supermix (172-5122; Bio-Rad, Richmond, CA, USA), and the cycles were measured using a CFX96 real-time PCR detection system (Bio-Rad). Primer sequences are listed in Table S1, and data are presented as fold change using the 2^-△△Ct^ method 56.

### Enzyme-linked immunosorbent assay (ELISA) analysis

The neurotrophin secretion of RSCs on the different hydrogels was evaluated with NGF, BDNF, and CNTF ELISA kits (Meimian Biotechnology, Yancheng, Jiangsu, China). Peptide solution (200 μL) was loaded directly into the wells of a 24-well plate, followed by the addition of 400 μL of culture medium onto the peptide solution and incubation for 15 min at 37 °C. After gelation, the medium was removed and changed at least twice in order to equilibrate the hydrogel to physiological pH. RSC suspension was added on the surface of the hydrogel (5 × 10^4^ cells/well, n = 4). After 3 days, the cell supernatant was extracted and used for the experiment according to the manufacturer's instructions. Subsequently, the absorbance values of the treated Microlon ELISA plates were measured by a microplate reader (Infinite F50, Tecan, Männedorf, Switzerland).

### Animal procedures

The experimental procedures involving animals were performed according to the Guide for the Care and Use of Laboratory Animals from the Chinese Ministry of Public Health and the United States National Institutes of Health. A total of 48 healthy male Sprague-Dawley rats (8 weeks old, 200-250 g) were provided by and maintained at the Experimental Animal Center of the Chinese PLA General Hospital. Rats were randomly divided into six groups (n = 8 rats/group): the hollow chitosan nerve conduit group (Hollow), the chitosan nerve conduit filled with RAD/IKV hydrogel (RAD/IKV), RAD/RGI hydrogel (RAD/RGI), RAD/IKV-GG-RGI hydrogel (RAD/IKV-GG-RGI), or RAD/IKV/RGI hydrogel (RAD/IKV/RGI) group, and the autologous nerve graft group (Autograft). We did not include pure RAD hydrogel as a separate group because previous study showed that unmodified RAD hydrogel had a poorer effect than functionalized peptide hydrogels 36. During surgery, rats were anesthetized via injection of 3% sodium pentobarbital solution (2.5 mg/100 g body weight), and the hair of the right femur was removed. The sciatic nerve of the right hind leg was exposed after making a skin incision and splitting the muscles, and a segment of the sciatic nerve was removed to leave a 10-mm-long gap after retraction of the nerve ends. The gaps were then bridged by different nerve conduits or autologous nerve according to group, and the muscle and skin were sutured. In the Hollow group, an empty chitosan tube was placed between the two ends of the transected nerve. In the RAD/IKV, RAD/RGI, RAD/IKV-GG-RGI, and RAD/IKV/RGI groups, the peptide solution was first injected into the lumen of the chitosan tube, and the tubes were then slowly submerged in DMEM for 15 min and transferred to physiological saline for use. In the Autograft group, a 10-mm transected segment was cut and reversed. All rats were housed and fed a standard diet with *ad libitum* access to food and water and monitored for any changes.

### Relative gene expression at the lesion site

At 1-week post-operation, the lesions of the rat sciatic nerve were harvested from all groups (n = 3/group), and the defect areas of tissue-containing conduits were cut and transferred immediately into liquid nitrogen in preparation for RNA extraction. qRT-PCR was performed, as described, using primers listed in **Table S1**.

### Histological assessment of regenerated nerves

At 12 weeks after surgery, five of the remaining rats in each group were euthanized, and the conduit samples with the tissue were harvested. The distal end of the regenerated nerves (**Figure S13**) was removed and fixed in pre-cooled 2.5% glutaraldehyde for 3 h, post-fixed with 1% osmium tetraoxide solution for 1 h, washed, dehydrated, embedded in Epon 812 epoxy resin (Fluka, Münster, Germany), and cut into 700-nm-thick semi-thin sections and 70-nm-thick ultrathin sections. The semi-thin sections were stained with 1% toluidine blue/1% borax solution and imaged under a model IX81 light microscope (Olympus, Tokyo, Japan). The total number of myelinated axons was counted and divided by the total area in 10 random fields of each sample, with the average as the density of myelinated axons for each group. The 70-nm-thick ultrathin sections were stained with lead citrate and uranyl acetate, and observed under a JEM-1400EX transmission electron microscope (TEM, JEOL Ltd., Tokyo, Japan). The TEM images were obtained from 10 random fields of each sample to determine the thickness of myelin sheaths, the diameter of myelinated nerve fibers, and the ratio between the mean diameter/perimeter of an axon and the mean diameter/perimeter of the fiber including myelin (g-ratio based on diameter/perimeter) using Image Pro Plus 6.0 software (Media Cybernetics).

### Electrophysiological recovery

At 12 weeks after surgery, the sciatic nerves at the injury site were re-exposed under deep anesthesia with sodium phenobarbital for electrophysiological studies (n = 5/group). Electric stimulation (3 mA) was applied between the proximal and distal nerve stumps, and compound muscle action potentials (CMAPs) were recorded at the target gastrocnemius muscle using a recording electrode. The latency and peak amplitude of the CMAPs were calculated and compared between different groups.

### Functional recovery of target gastrocnemius muscle

At 12 weeks after surgery, the gastrocnemius muscles harvested from the injured and contralateral sites (n = 5/group) were immediately weighed and then fixed in 4% paraformaldehyde at 4 °C for 7 days. The muscle samples were transversely cut into 7-μm-thick paraffin sections and subjected to Masson's trichrome staining. For each sample, images were obtained from 10 randomly chosen fields with Zeiss Axio Scan.Z1 Scanner (Carl Zeiss). The muscle wet weight ratio and cross-sectional area of the muscle fibers were quantitatively analyzed with Image Pro Plus 6.0 software (Media Cybernetics).

### Motor functional analysis

The CatWalk XT 9.0 gait analysis system (Noldus, Wageningen, The Netherlands) was used to evaluate the motor functional recovery at 2, 4, 6, 8, 10, 12 weeks after surgery. Rats (n = 5/group at each time point) were placed on the right side of the runway comprising a glass surface and a black plastic wall. Each run of the rat was captured by a high-speed camera located under the runway, and the stand time, contact area and intensities of the right injured hind paw (RH) and the normal left hind paw (LH) were recorded. Sciatic function index (SFI) was calculated according to the following formula:

SFI=109.5((ETS-NTS)/NTS)-38.3((EPL-NPL)/NPL)+13.3((EIT-NIT)/NIT)-8.8

where ETS refers to the experimental toe spread, NTS refers to the normal toe spread, EPL refers to the experimental print length, NPL refers to normal print length; EIT refers to experimental inter toe spread, and NIT refers to normal inter toe spread.

### Statistical analysis

All data are presented as mean ± standard deviation (SD). For material characterization and* in vitro* study, each experiment was conducted independently at least three times. The normality test was performed using Kolmogorov-Smirnov test in SPSS (v.23.0; IBM corp., Armonk, NY, USA). Statistical analysis of data conforming to normal distribution was carried out using one-way analysis of variance (ANOVA), followed by Tukey's post hoc test (equal variances) or Dunnett's T3 post hoc test (unequal variances). Data inconsistent with normal distribution were statistically analyzed via non-parametric method in conjunction with Bonferroni post hoc test. A *P* < 0.05 was considered statistically significant.

## Results

### Microstructure and mechanical properties of hydrogels

**Figure 1:**
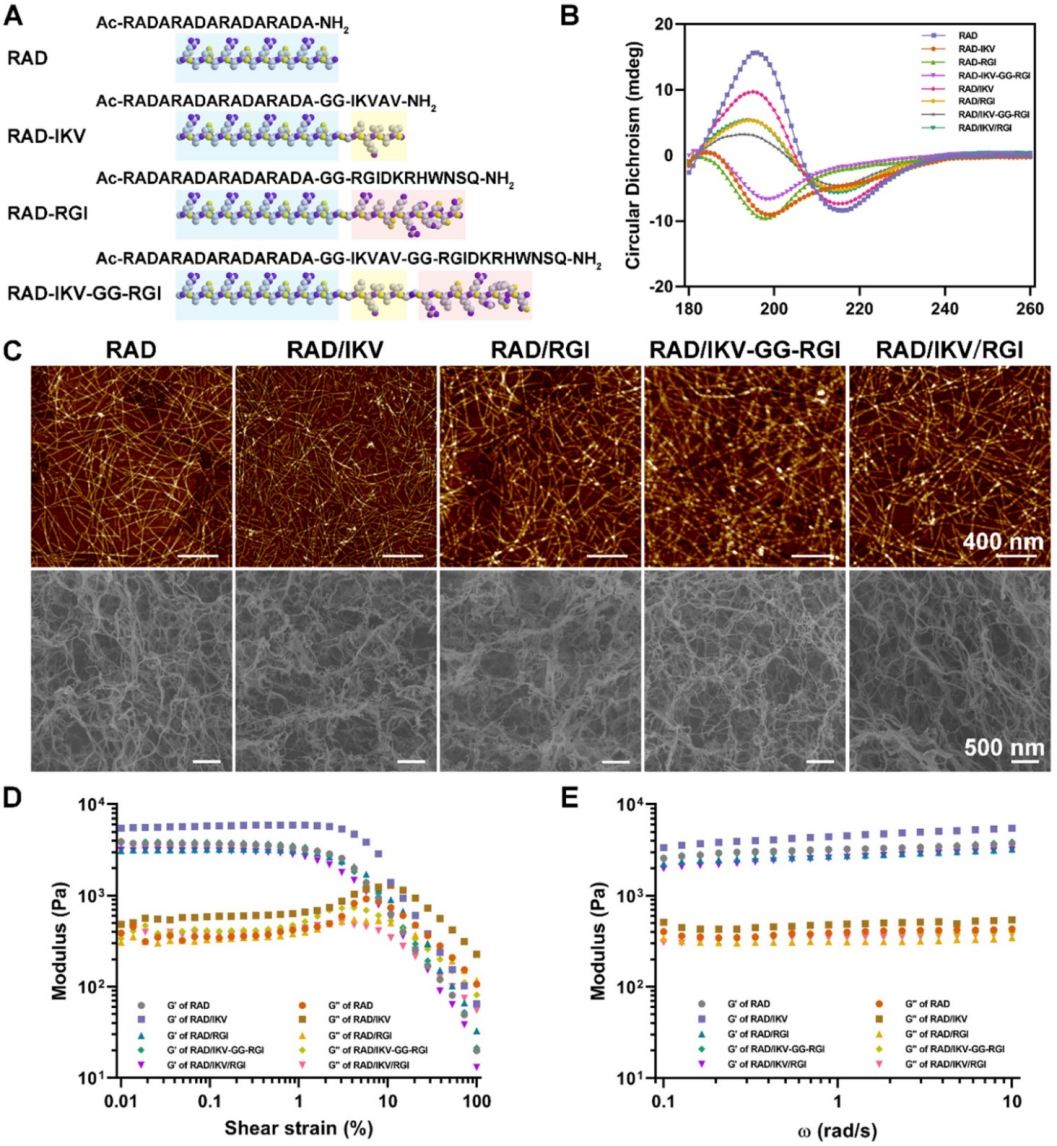
Characterization of the SAP solutions and hydrogels. (**A**) Sequences of the functionalized self-assembling peptides. (**B**) Typical CD spectra of RAD, RAD-IKV, RAD-RGI, RAD-IKV-GG-RGI, RAD/IKV, RAD/RGI, RAD/IKV-GG-RGI, and RAD/IKV/RGI solutions. (**C**) AFM images of the peptide solutions and SEM morphologies of the hydrogels. (**D**) Rheological characterization of the peptide hydrogels by strain sweep studies, where the storage modulus (G') and loss storage modulus (G'') were recorded as a function of oscillation strain (%). (**E**) Dynamic frequency sweep test (0.1-10 rad/s at 0.5% strain) of the peptide hydrogels.

We prepared the series of peptides using solid phase peptide synthesis. The physicochemical properties of self-assembling backbone and functional motifs were shown in **Table S2**. The common RADARADARADARADA backbone of the peptides used in this study contains a hydrophobic side with an array of alanine (A) and a side with alternating negatively charged aspartic acids (D) and positively charged arginine (R), allowing the peptide to form a stable β-sheet structure via both electrostatic and hydrophobic interactions 13. The functionalized peptides RAD-IKV, RAD-RGI, and RAD-IKV-GG-RGI were synthesized by directly extending the C-terminus of RAD, with the two glycine residues between the backbone and the functional motifs designed to maintain flexibility. Similarly, the glycine residues between IKVAV sequence and RGI sequence in RAD-IKV-GG-RGI peptide were used to reduce the interference between the two functional segments (**Figure 1A**). The characterization of peptide synthesis via high performance liquid chromatography and electrospray ionization mass spectrometry showed that the peptides were synthesized successfully (**Figure S1-S8**). Then we analyzed the secondary structure of the peptides using circular dichroism. As shown in **Figure 1B**, the CD spectra of pure RAD-IKV, RAD-RGI, and RAD-IKV-GG-RGI suggested disordered structures with a negative band at ~200 nm. After mixing with RAD, the spectra of RAD/IKV, RAD/RGI, RAD/IKV-GG-RGI, and RAD/IKV/RGI mixtures showed a typical pattern for a β-sheet structure with a positive band at 195 nm and a negative band at 216 nm. Increases in the chain length of the functional motifs decreased the intensity of the CD spectra relative to that for pure RAD, with RAD/IKV-GG-RGI showing the lowest intensity and RAD the highest. Additionally, RAD/IKV/RGI presented a similar CD spectrum to RAD/RGI, indicating that the longer motif played a primary role in weakening the self-assembly behavior of the mixed peptide. The secondary structure fractions of different peptides were calculated according to the CD spectra and listed in **Table S3**. In pure RAD-IKV, RAD-RGI, and RAD-IKV-GG-RGI solutions, β-turn structure and unordered structure accounted for the highest proportion, which were 71.8%, 87.2% and 69.5%, respectively; however, few β-sheet structure was observed, conforming to the spectra results. The result indicated that the pure functionalized peptides underwent a weak self-assembling process because the formation of β-sheet structure was somewhat affected by the functional motifs. The pure solutions of RAD-IKV, RAD-RGI, and RAD-IKV-GG-RGI could hardly form hydrogels, which were not used in the following experiments. In RAD/IKV, RAD/RGI, RAD/IKV-GG-RGI, and RAD/IKV/RGI solutions, β-sheet structure accounted for the highest proportion, which were 52.2%, 47.9%, 40.3% and 45.9%, respectively, while the β-sheet content in RAD solution was 46.4%. The result indicated that RAD-IKV could enhance the β-sheet formation while RAD-IKV-GG-RGI hindered the β-sheet formation. Additionally, RAD-RGI exhibited little influence on the formation of β-sheet structure.

AFM examination of the nanostructures formed by the functionalized peptide solutions (**Figure 1C**) revealed uniform and interweaved long nanofibers in both RAD and the functionalized peptide mixtures, suggesting that the functionalized peptides interacted with RAD and incorporated into the nanofibers. The nanofiber widths of different peptides were calculated form molecular models and measured from AFM images (**Table S4**). We assumed that the functional motifs extended out of the self-assembling backbone to widen the nanofibers and the peptides co-assembled evenly in the solution. The nanofibers of RAD (15.4 ± 2.1 nm) were thinner than those of other peptides in theory and measurement. Among four functionalized peptide groups, RAD/IKV had the thinnest nanofibers (18.1 ± 1.9 nm), while RAD/IKV-GG-RGI had the widest nanofibers (29.6 ± 2.2 nm). The width of nanofibers in RAD/RGI (23.0 ± 1.5 nm) and RAD/IKV/RGI (21.6 ± 0.9 nm) groups was almost the same, indicating that the longer motif determined the nanofiber width. The measured width was consistent with the adjusted theoretical width, confirming the co-assembling model of different peptides. Peptide solutions were subsequently equilibrated in DMEM at 37 °C for 15 min and formed hydrogels. SEM imaging confirmed the presence of self-assembled nanofibers of the peptide hydrogels (**Figure 1C**), suggesting that the functionalized peptides possessed the same nanostructure as the RAD hydrogel, and offered a 3D microenvironment for cell growth by mimicking the ECM. The pore sizes of different self-assembling peptide hydrogels were measured according to SEM images (**Figure S9**). The pores in the hydrogels were mostly in the size of 10 - 200 nm, in which those ranging from 25 nm to 100 nm accounted for a large proportion. The mean diameter of pores was not significantly different among all the hydrogels, which was around 80 nm.

We then performed rheological measurements of the mechanical properties of the hydrogels. The storage modulus (G') positively correlates with the mechanical rigidity, and the loss modulus (G'') is related to the viscous properties of the hydrogel. In stress/strain sweep test, the G' of each hydrogel remained constant when the shear strain was between 0.01% and ~1% and decreased dramatically after 3%, whereas G'' showed the same trend at <1% but increased from 1% to ~5%, followed by a steady decrease, indicating that the limits of the linear viscoelastic region reached at around 1% strain (**Figure 1D**). To determine hydrogel stability, we performed a frequency sweep test at a constant shear strain of 0.5%. The hydrogels exhibited steady G' and G'' values at frequencies ranging from 0.1 rad/s to 10 rad/s with G' values (~3.0 kPa) obviously larger than G'' values (~0.4 kPa), indicating that the hydrogels exhibited gel-like and elastic properties with elasticity approaching that of nerve tissue matrix (**Figure 1E**) 57. Additionally, the two moduli of RAD/IKV hydrogel were significantly higher than those of the other hydrogels, indicating the hydrogel might have a higher stiffness than other hydrogels (**Figure S10**).

### Myelination of RSCs on hydrogels *in vitro*

**Figure 2:**
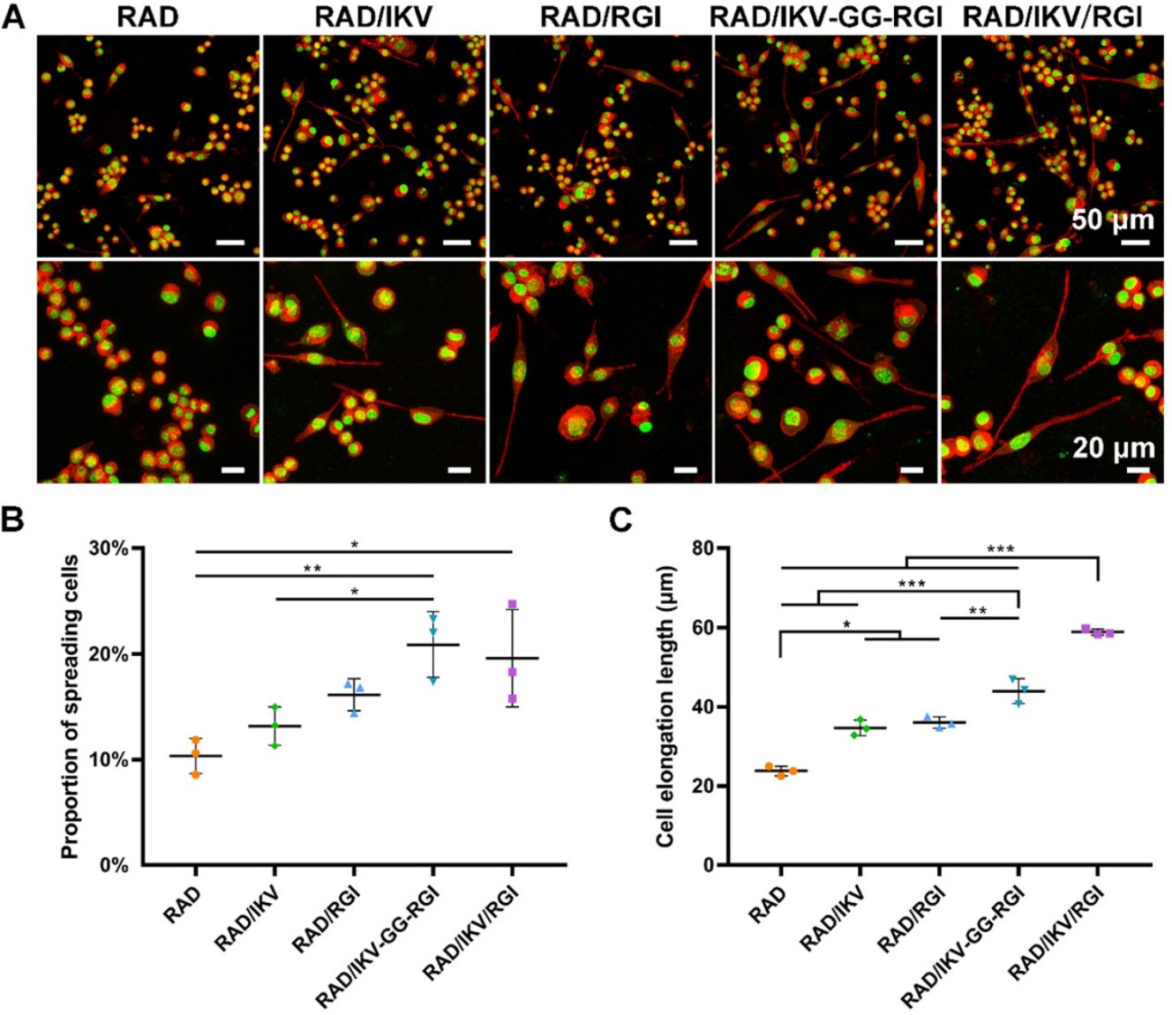
RSC adhesion and spreading behavior on the surface of different SAP hydrogels. (**A**) Representative fluorescence microscopy images of RSCs on different peptide scaffolds following a 7-day culture. Cells were stained with rhodamin-phalloidin for β-actin (red) and SYTOX Green for nuclei (green). (**B**) The proportion of RSCs spreading on the hydrogels. (**C**) The cell elongation length of RSCs adhered to the hydrogels. Values are represented as mean ± SD. Statistical analysis was carried out using one-way analysis of variance (ANOVA), followed by Tukey's post hoc test (equal variances) or Dunnett's T3 post hoc test (unequal variances). *P < 0.05, **P < 0.01, and ***P < 0.001 (n = 3).

SCs play an important role in the PNS and reconstruction of regenerated nerves via myelination. The RSCs had a significantly faster proliferation on RAD/IKV-GG-RGI and RAD/IKV/RGI hydrogels than other hydrogels, indicating that the dual-functionalized hydrogels could enhance the proliferation of RSCs (*P* < 0.01, **Figure S11**). To evaluate the effect of the hydrogels on RSC adhesion and spreading, the cell morphologies were observed under the confocal microscope after a 7-day incubation of RSCs on hydrogels (**Figure 2A**). SCs undergo morphological transitions as they develop from immature to myelinating glial cells 54, 58. The polarized and elongated morphology is closely related to the myelination of SCs, which was commonly evaluated in studies concerning SCs 55, 59, 60. In our study, we used these indices to identify the myelination of SCs on different hydrogels. RSCs exhibited a spherical morphology on the RAD hydrogel but tended to spread on the RAD/IKV, RAD/RGI, RAD/IKV-GG-RGI, and RAD/IKV/RGI hydrogels along with formation of a long protuberance. Additionally, the proportions of spreading cells on the RAD/IKV-GG-RGI (20.89% ± 3.10%) and RAD/IKV/RGI (19.60% ± 4.59%) hydrogels were significantly higher than that on the RAD hydrogel (10.35% ± 1.66%) (*P* < 0.01 or *P* < 0.05), with both hydrogels also showing higher mean values of spreading cells than RAD/IKV (13.18% ± 1.83%) and RAD/RGI (16.15% ± 1.51%) hydrogels (**Figure 2B**). The cell elongation lengths on RAD/IKV-GG-RGI (43.97 ± 3.13 μm) and RAD/IKV/RGI (58.90 ± 0.77 μm) hydrogels were significantly longer than the other hydrogels (*P* < 0.01 or *P* < 0.001), and, notably, cells on the RAD/IKV (34.68 ± 1.99 μm) and RAD/RGI (36.02 ± 1.44 μm) hydrogels showed significant longer elongation lengths than those on the RAD hydrogel (23.78 ± 1.19 μm) (*P* < 0.05, **Figure 2C**), indicating promoted overall cell adhesion and myelination on the functionalized peptide hydrogels.

**Figure 3:**
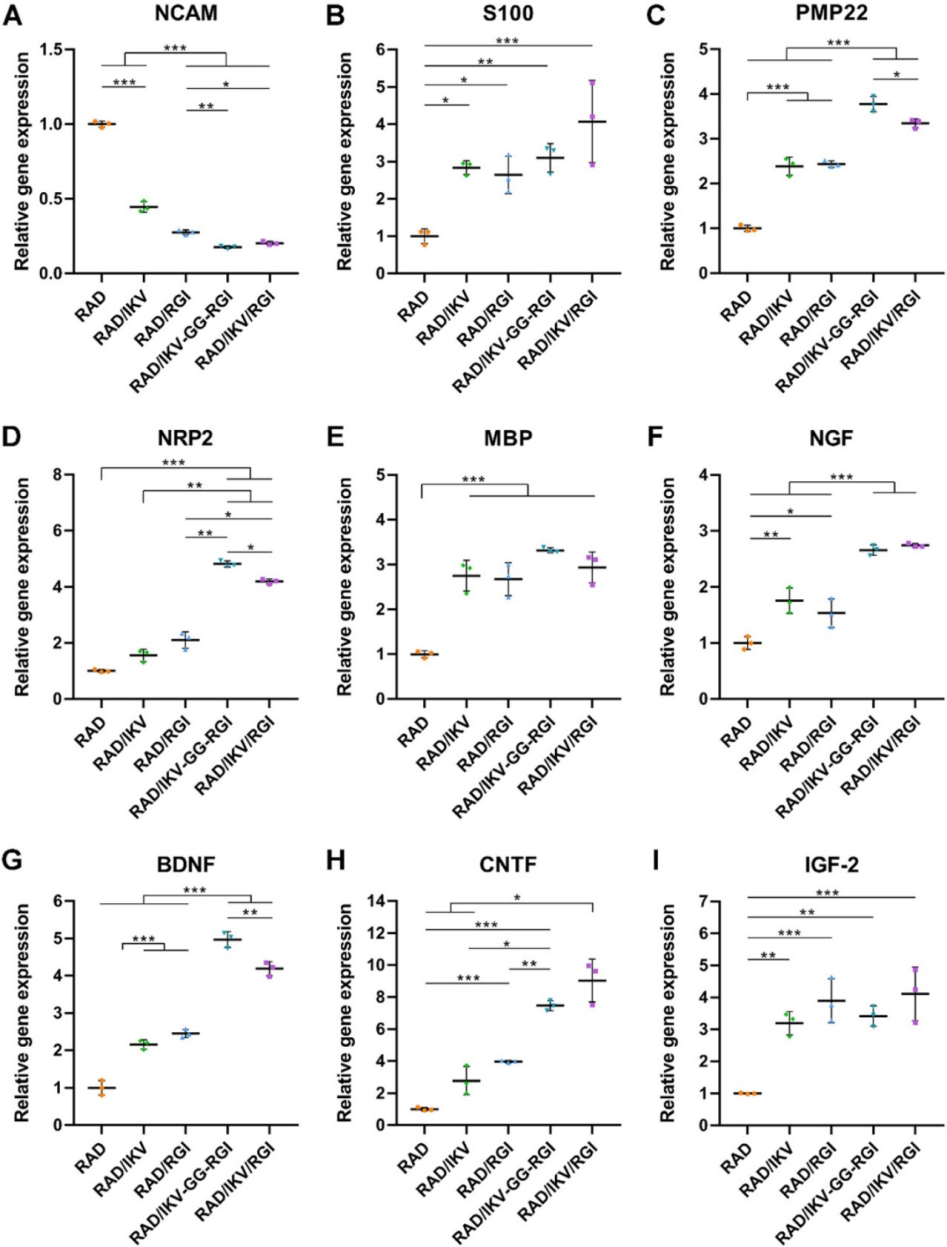
Gene expression in RSCs cultured on the hydrogels for 7 days. qPCR analysis of NCAM (**A**), S100 (**B**), PMP22 (**C**), NRP2 (**D**), MBP (**E**), NGF (**F**), BDNF (**G**), CNTF (**H**) and IGF-2 (**I**). Values are represented as mean ± SD. Statistical analysis was carried out using one-way analysis of variance (ANOVA), followed by Tukey's post hoc test (equal variances) or Dunnett's T3 post hoc test (unequal variances). *P < 0.05, **P < 0.01, and ***P < 0.001 (n = 3).

**Figure 4:**
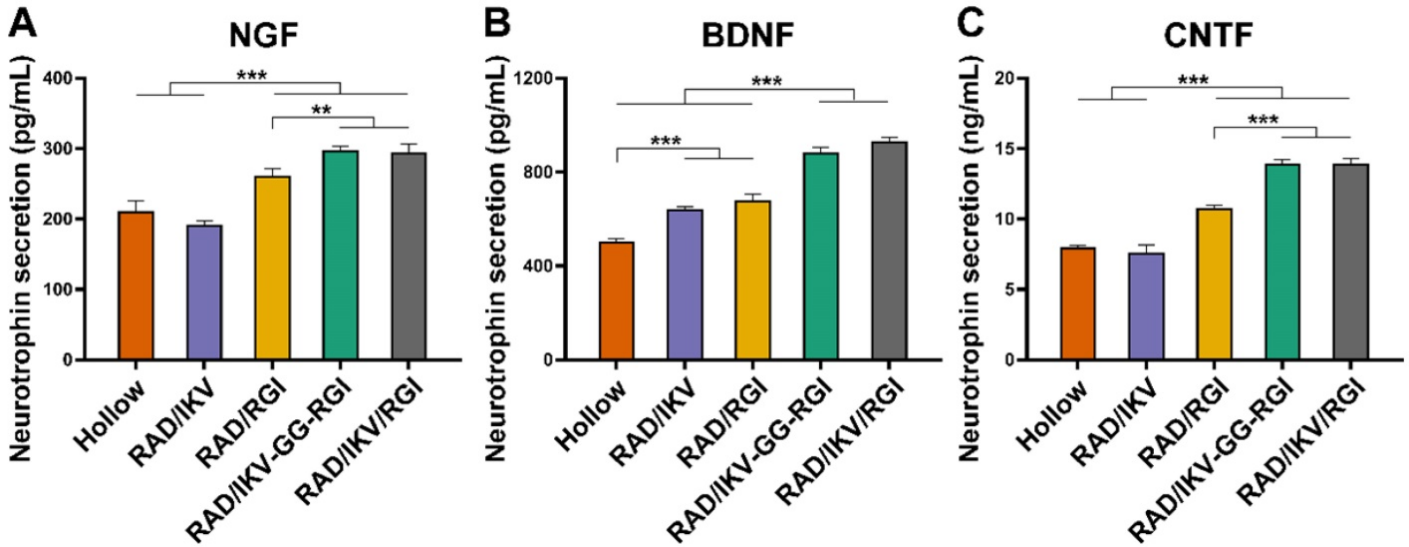
Neurotrophin secretion of NGF (A), BDNF (B), and CNTF (C) of RSCs on different peptide hydrogels following a 3-day culture. Values are represented as mean ± SD. Statistical analysis was carried out using one-way analysis of variance (ANOVA), followed by Tukey's post hoc test (equal variances) or Dunnett's T3 post hoc test (unequal variances). *P < 0.05, **P < 0.01, and ***P < 0.001 (n = 4).

Immature SCs usually express neuronal cellular adhesion molecules (NCAM) and p75 during their development, whereas myelinating SCs reduce NCAM expression and increase levels of myelinating proteins and neurotrophin secretion at the myelination stage 61. Therefore, we examined the expression of nine related genes from RSCs, including NCAM, S100, peripheral myelin protein (PMP22), myelin basic protein (MBP), neuropilin 2 (NRP2), NGF, BDNF, CNTF, and insulin-like growth factor-2 (IGF-2), by culturing cells on different hydrogels for 7 days, with RAD used as a control group. The results showed that NCAM expression on the RAD hydrogel was significantly higher than that on the other hydrogels (*P* < 0.001), whereas RSCs on the RAD/IKV-GG-RGI and RAD/IKV/RGI hydrogels showed significantly lower levels of NCAM expression relative to those on the RAD/IKV and RAD/RGI hydrogels (*P* < 0.05, **Figure 3A**). The S100 expression on the RAD hydrogel was significantly lower than that on the other hydrogels (*P* < 0.05, **Figure 3B**). The expression of myelin genes in RSCs, including PMP22 and NRP2, on the RAD/IKV-GG-RGI and RAD/IKV/RGI hydrogels was significantly higher than that in the other three groups, which was consistent with morphologic observations (*P* < 0.05, **Figure 3C-D**). The MBP expression on the functionalized peptide hydrogels was significantly higher than that on the RAD hydrogel (*P* < 0.001, **Figure 3E**). Additionally, the gene expression of NGF, BDNF, and CNTF on RAD/IKV-GG-RGI and RAD/IKV/RGI hydrogels was significantly higher than that on the other three hydrogels (*P* < 0.05), whereas the expression of the three neurotrophic factors on RAD/IKV and RAD/RGI hydrogels was significantly higher than that on the RAD hydrogel (*P* < 0.05), indicating that IKVAV and RGI motifs enhanced the neurotrophin secretion of RSCs, respectively and synergistically (**Figure 3F-H**). Furthermore, the expression of IGF-2 was significantly increased on functionalized hydrogels compared with the RAD hydrogel (*P* < 0.01, **Figure 3I**). We then evaluated the neurotrophin secretion at the protein level. The secretion of NGF, BDNF, and CNTF of RSCs on RAD/IKV-GG-RGI and RAD/IKV/RGI hydrogels after 3-day culture was also significantly higher than that on the other three hydrogels (*P* < 0.01), which was consistent with the gene expression (**Figure 4A-C**). Additionally, the secretion of NGF and CNTF of RSCs on RAD/RGI hydrogel was significantly higher than those on RAD and RAD/IKV hydrogels (*P* < 0.001), indicating that RGI motif showed a high ability to promote these neurotrophins. The secretion of BDNF on RAD/IKV and RAD/RGI hydrogels was significantly higher than that on RAD hydrogel (*P* < 0.001), suggesting that the single motif could also promote the neurotrophin secretion, which was in accordance with previous study 62. The results indicated that the dual-functionalized peptides could significantly enhance the neurotrophin secretion.

### Relative gene expression in nerve grafts

**Figure 5:**
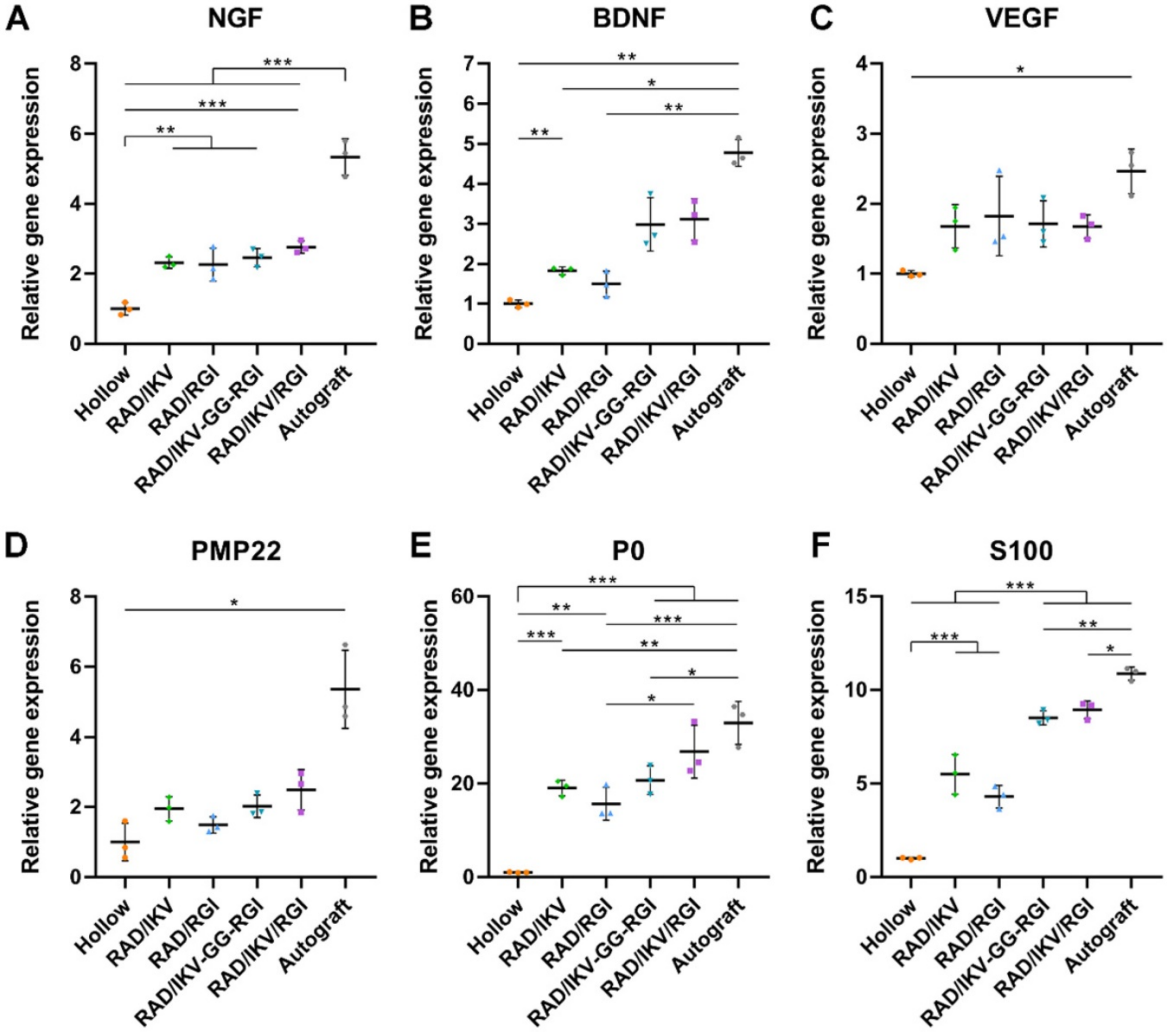
Relative gene expression in nerve grafts after 7 days. qRT-PCR analysis of NGF (**A**), BDNF (**B**), VEGF (**C**), PMP22 (**D**), P0 (**E**), S100 (**F**). Values are represented as mean ± SD. Statistical analysis was carried out using one-way analysis of variance (ANOVA), followed by Tukey's post hoc test (equal variances) or Dunnett's T3 post hoc test (unequal variances). *P < 0.05, **P < 0.01, and ***P < 0.001 (n = 3).

Six groups of nerve grafts (Hollow, RAD/IKV, RAD/RGI, RAD/IKV-GG-RGI, RAD/IKV/RGI, and Autograft) were implanted to bridge the 10-mm gap in the transected sciatic nerve in rats (**Figure S12A-B**). To investigate the effect of the peptide hydrogels on nerve regeneration* in vivo*, the nerve grafts of all groups were harvested at 1-week post-operation (**Figure S12C**), and the gene expression of neurotrophic factors (NGF and BDNF), vascular endothelial growth factor (VEGF), and myelin genes inside the nerve graft during the early stage of nerve regeneration were evaluated by qRT-PCR. Generally, the Autograft group showed the highest expression of regeneration-related genes, whereas the Hollow group exhibited the lowest expression. The expression of NGF and BDNF in the groups with functionalized hydrogels was significantly higher than that in the Hollow group (*P* < 0.05, **Figure 5A-B**). However, the expression of VEGF and PMP22 in functionalized groups was promoted a little compared with that in the Hollow group without significant difference, approaching that in the Autograft group (*P* > 0.05, **Figure 5C-D**). Additionally, functionalized peptide hydrogels showed significantly increased gene expression of P0 and S100 than that associated with use of the hollow chitosan tube (*P* < 0.01,** Figure 5E-F**). Moreover, P0 expression in the RAD/IKV/RGI group was comparable to the Autograft group (*P* > 0.05) and significantly higher than that in the RGI group (*P* < 0.05). S100 expression in the RAD/IKV-GG-RGI and RAD/IKV/RGI groups was significantly higher than that in the Hollow, RAD/IKV, and RAD/RGI groups (*P* < 0.001), indicating a synergistic effect of IKVAV and RGI motifs. These results suggested that dual-functionalized peptide hydrogels promoted the growth factor secretion-related and myelination-related gene expression in the early stage of nerve grafting along with specifically higher levels of P0 and S100 that had a close relationship with SC myelination and development, thus enhancing the regeneration.

### Histological recovery of regenerated nerves

**Figure 6:**
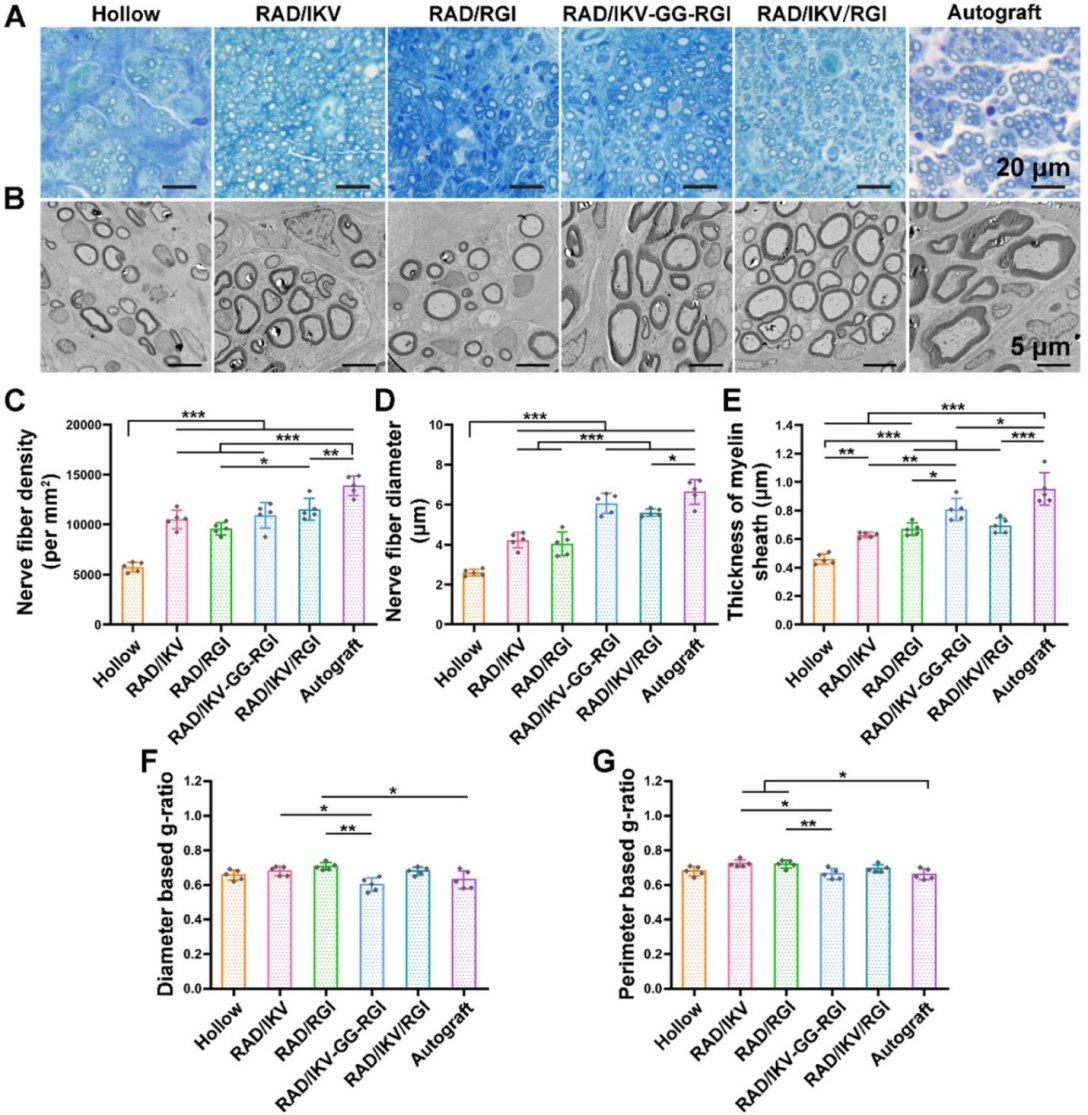
Evaluation of regenerated nerve fibers obtained from the distal sites of nerve grafts at 12 weeks after surgery. (**A**) Toluidine blue-stained transverse sections of the harvested nerve grafts. (**B**) Transmission electron micrographs of the regenerated nerves. Histomorphometric analysis was performed by calculating the density of myelinated nerve fibers (**C**), the diameter of the myelinated axons (**D**), the thickness of the myelin sheath (**E**), diameter-based g-ratio (**F**), and perimeter-based g-ratio (**G**). Values are represented as mean ± SD. Statistical analysis was carried out using one-way analysis of variance (ANOVA), followed by Tukey's post hoc test (equal variances) or Dunnett's T3 post hoc test (unequal variances). *P < 0.05, **P < 0.01 and ***P < 0.001 (n = 5).

At 12 weeks after surgery, the cross-sections of the distal end of the regenerated nerves were observed under a light microscope and by TEM (**Figure S13**). Histological analysis of the regenerated tissue by toluidine blue staining showed that uniform myelinated nerve fibers had grown across the entire gap to the distal end in all groups (**Figure 6A**). Quantitative calculation of myelinated nerve fiber density according to toluidine blue staining pictures in all groups revealed that the Autograft group showed the highest density (13886.90 ± 980.25 /mm^2^), while the densities of the RAD/IKV-GG-RGI (10934.23 ± 1270.93 /mm^2^) and RAD/IKV/RGI (11522.98 ± 1079.32 /mm^2^) groups were lower than that of the Autograft group (*P* < 0.01). The Hollow group showed the significantly lower density (5749.22 ± 221.01 /mm^2^) than the other groups (*P <* 0.001). Additionally, the fiber density of the RAD/IKV/RGI group was significantly higher than that of the RAD/RGI group (9585.01 ± 598.07 /mm^2^) (*P <* 0.05, **Figure 6C**).

Axonal morphologies at the distal end showed more densely packed nerve fibers in the RAD/IKV-GG-RGI and RAD/IKV/RGI groups than in the RAD/IKV and RAD/RGI groups, with all functionalized groups much better than the Hollow group (**Figure 6B**). The mean diameter of myelinated nerve fibers and the thickness of the myelin sheath according to TEM analysis revealed a larger diameter of the myelinated nerve fibers and a thicker myelin sheath in the dual-functionalized groups compared with those in Hollow, RAD/IKV, and RAD/RGI groups, indicating better re-myelination (*P <* 0.05, **Figure 6D-E**). The nerve fiber diameter and myelin sheath thickness in RAD/IKV and RAD/RGI groups were significantly higher than those in Hollow group (*P <* 0.01), indicating the promotion effect on myelination of single functionalized peptides. Additionally, the diameter-based g-ratio and perimeter-based g-ratio were evaluated, which are indicative of the degree of fiber myelination (**Figure 6F-G**). For normal peripheral nerve, the optimal value of g-ratio is found to approach 0.6 theoretically and experimentally 63, 64. The average values of diameter-based and perimeter-based g-ratios in all groups fluctuate up and down at 0.6, indicating that the myelination of the nerve fibers was generally complete. The g-ratios of Hollow, RAD/IKV-GG-RGI, and RAD/IKV/RGI groups were not significantly different from those of Autograft group (*P >* 0.05). Additionally, the two g-ratios of RAD/IKV-GG-RGI group were significantly lower than those of RAD/IKV (*P <* 0.05) and RAD/RGI groups (*P <* 0.01), suggesting an improved effect of RAD/IKV-GG-RGI hydrogel on myelination. The diameter-based g-ratio of RAD/RGI group and the perimeter-based g-ratio of RAD/IKV and RAD/RGI groups were significantly higher than those of Autograft, respectively, indicating a slower but potential myelination in single functionalized peptides. Overall, we identified more thick regenerated myelinated nerves in the RAD/IKV-GG-RGI and RAD/IKV/RGI groups than in other groups except for the Autograft group, suggesting improved myelination induced by co-effects of IKVAV and RGI motifs.

### Electrophysiological recovery

**Figure 7:**
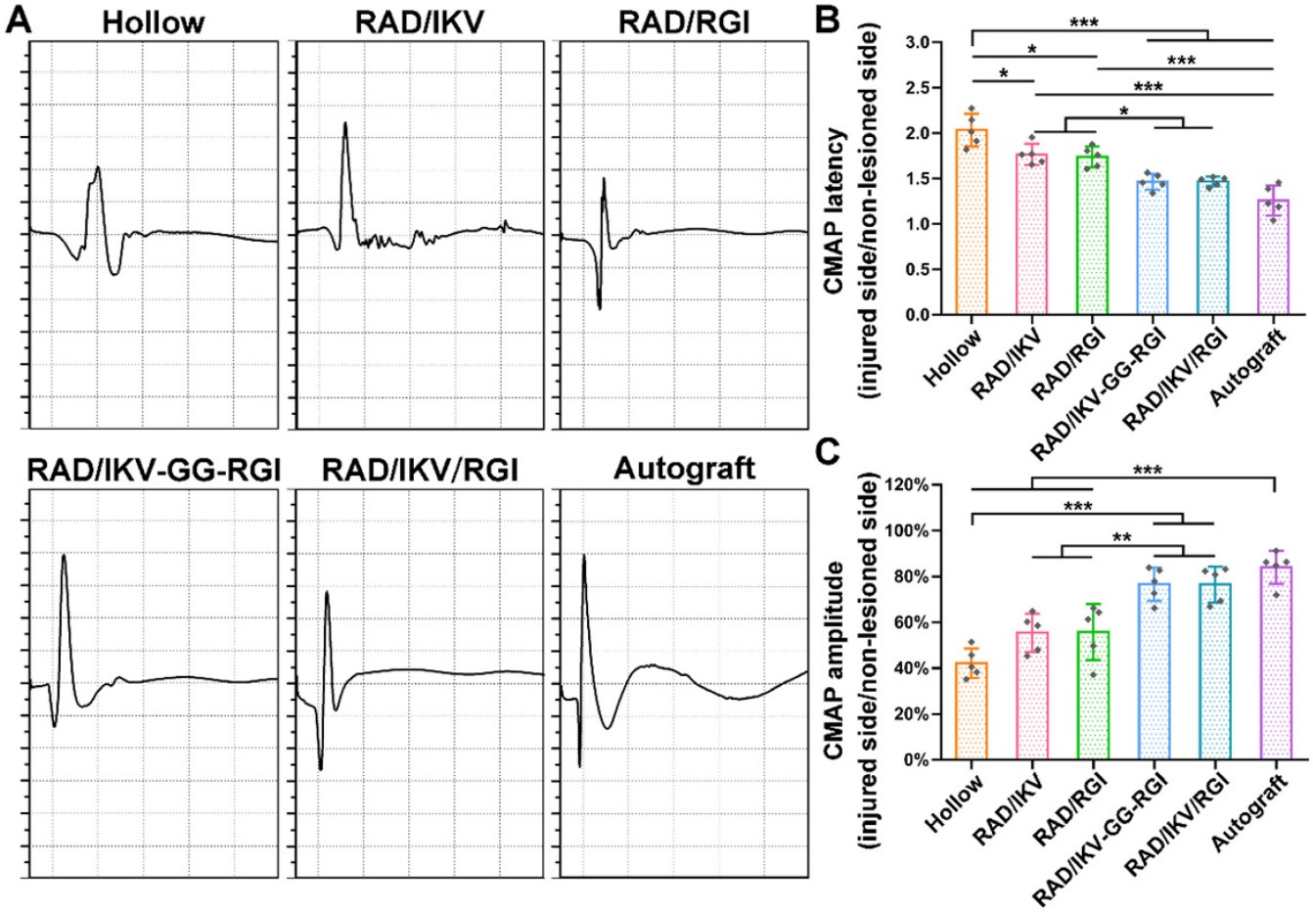
Electrophysiological data acquired at 12 weeks after surgery. (**A**) Representative CMAP recordings from the injured sides of rats in each group. Sciatic CMAP amplitude (**B**) and CMAP latency (**C**) of the injured side of rats compared with non-lesioned side in each group. Values are represented as mean ± SD. Statistical analysis was carried out using one-way analysis of variance (ANOVA), followed by Tukey's post hoc test (equal variances) or Dunnett's T3 post hoc test (unequal variances). *P < 0.05, **P < 0.01, and ***P < 0.001 (n = 5).

The recovery of nerve conduction in each group was then evaluated by electrophysiological examination according to CMAP index, including CMAP amplitude reflecting the number of innervated muscle fibers and CMAP latency to evaluate the degree of myelination. The typical CMAP curves of each group at 12 weeks after surgery showed the CMAP latency in the RAD/IKV-GG-RGI, RAD/IKV/RGI and Autograft groups was obviously lower than the other three groups, while the CMAP amplitude was obviously higher (**Figure 7A**). The quantitative analysis showed that the ratio of CMAP latency between the injured side and non-lesioned side in the RAD/IKV-GG-RGI (1.46 ± 0.09) and RAD/IKV/RGI (1.47 ± 0.05) groups was significantly lower than that in the Hollow (2.03 ± 0.18, *P <* 0.001), RAD/IKV (1.77 ± 0.12, *P <* 0.05), and RAD/RGI groups (1.74 ± 0.11, *P <* 0.05), with no significant difference from that of the Autograft (1.26 ± 0.17, *P* > 0.05), indicating that the myelination degree of the dual-functionalized peptide group was comparable to that of the Autograft group at 12 weeks (**Figure 7B**). Additionally, the ratio of CMAP amplitude between the injured side and non-lesioned side in the RAD/IKV-GG-RGI (76.6 ± 7.3%) and RAD/IKV/RGI (76.5 ± 7.7%) groups was significantly higher than that in the Hollow (42.2 ± 6.4%, *P <* 0.001), RAD/IKV (55.4 ± 8.3%, *P <* 0.01), and RAD/RGI (55.7 ± 12.2%, *P <* 0.01) groups, comparable to the Autograft group (84.0 ± 7.2%, *P >* 0.05) (**Figure 7C**). These results indicated that the IKVAV and RGI motifs synergistically promoted re-myelination and facilitated the electrophysiological recovery of the regenerated nerve. Moreover, the ratio of CMAP amplitude in the RAD/IKV and RAD/RGI groups were significantly better than those of Hollow group (*P <* 0.05), indicating the positive effects of LN and BDNF on recovered nerve activity, respectively.

### Functional recovery of target gastrocnemius muscle

**Figure 8:**
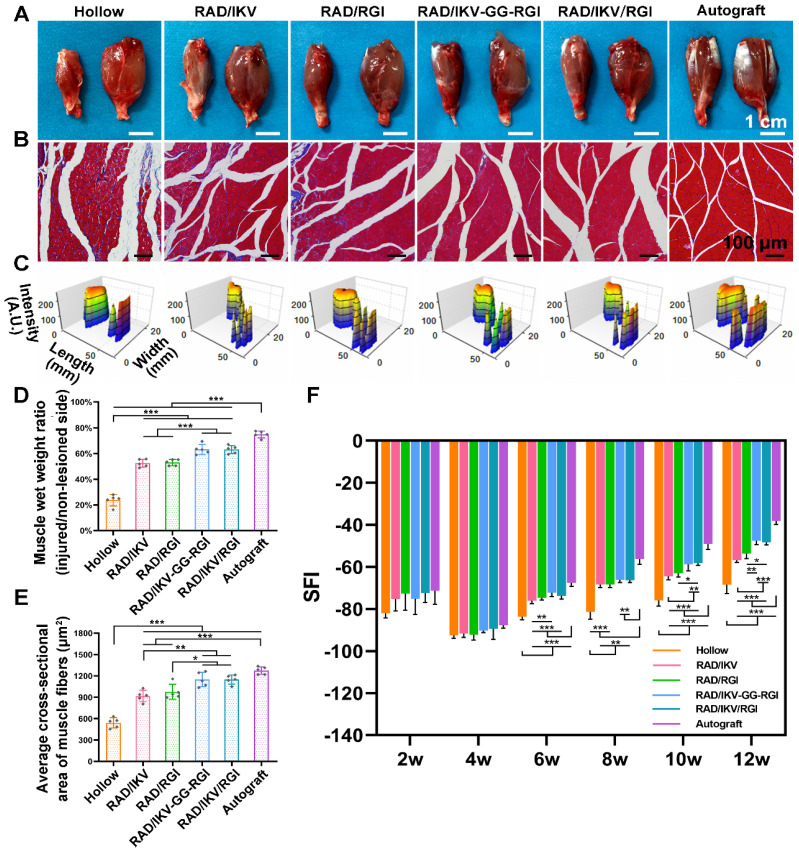
Functional recovery at 12 weeks after surgery. (**A**) Gross images of the isolated gastrocnemius muscles at 12 weeks. (**B**) Masson's trichrome staining of transverse sections of the muscles of the injured limbs. (**C**) 3D plantar pressure distributions of the hindlimbs of the injured side. Statistical analysis of muscle wet weight ratios of injured side to non-lesioned side (**D**), and the average cross-sectional areas of the muscle fibers (**E**). (**F**) SFI values over a 12-week observation period. Values are represented as mean ± SD. Statistical analysis was carried out using one-way analysis of variance (ANOVA), followed by Tukey's post hoc test (equal variances) or Dunnett's T3 post hoc test (unequal variances). *P < 0.05, **P < 0.01, and ***P < 0.001 (n = 5).

Target muscles gain re-innervation after motor nerve regeneration, with their morphology and motor endplate formation reflecting the functional outcomes. Nerve regeneration and the reconstruction of connections between nerves and target muscle allow muscles to gradually return to normal. At 12 weeks postoperatively, gastrocnemius muscles from both sides were harvested, weighed, and stained with Masson's trichrome at the transverse sections of the muscles from the injured limbs. The gross images of the harvested muscles showed that sciatic nerve injury caused muscle atrophy of the injured site and the size of the muscle had not recovered to normal at 12 weeks (**Figure 8A**). The muscle wet weight ratios at the injured side compared with the normal side in the RAD/IKV-GG-RGI (63.26% ± 3.93%) and RAD/IKV/RGI (63.13% ± 3.19%) groups were statistically higher than those in the Hollow (23.70% ± 4.47%), RAD/IKV (52.49% ± 3.06%), and RAD/RGI (53.00% ± 2.43%) groups (*P <* 0.001), but significantly lower than that in the Autograft group (74.89% ± 2.66%, *P <* 0.001) (**Figure 8D**). Additionally, staining results of the muscles and statistical analysis indicated significantly decreased collagen fibers (blue in the images) and significantly larger average cross-sectional area of gastrocnemius muscle fibers in the RAD/IKV-GG-RGI (1149.34 ± 102.36 μm^2^) and RAD/IKV/RGI (1149.10 ± 63.61 μm^2^) groups relative to the Hollow (540.84 ± 72.69 μm^2^), RAD/IKV (917.07 ± 80.63 μm2), and RAD/RGI (976.26 ± 105.07 μm^2^) groups (*P <* 0.05) and similar to results in the Autograft group (1274.74 ± 53.76 μm^2^, P > 0.05, **Figure 8B, E**). Furthermore, the muscle wet weight and cross-sectional area in the RAD/IKV and RAD/RGI groups were significantly higher than the Hollow group (*P <* 0.001).

The muscle atrophy leads to a morbid walking gait with the toes curling up, and the footprint returns to normal when the target muscles gain re-innervation and motor function recovers. At 12 weeks, the toe spread in the dual-functionalized peptide groups was obviously similar to that in the Autograft and better than those in the other groups, indicating a recovery in motor function (**Figure 8C**). SFI values were calculated to evaluate the functional recovery of the regenerated nerves. An SFI value of 0 represents normal motor function, whereas a value of -100 indicates complete dysfunction. After surgery, the SFI of rats in all groups followed the same trend, where the values initially decreased in 4 weeks and then increased as a result of successful nerve regeneration 65, 66. There was no significant difference among groups at 2 and 4 weeks after surgery (P > 0.05); however, after 4 weeks, the SFI values in the functionalized peptide groups displayed significant differences compared with those in the Hollow group (*P <* 0.01 or *P <* 0.001), whereas after 8 weeks, the SFI values in the RAD/IKV-GG-RGI and RAD/IKV/RGI groups were significantly higher than those in the Hollow, RAD/IKV, and RAD/RGI groups (*P <* 0.05) and were the closest to those in the Autograft group (**Figure 8F**). These results suggested that the dual-functionalized peptide hydrogel could significantly improve the functional recovery after sciatic nerve injury.

## Discussion

PNI is a significant cause of morbidity and lifelong disability, despite surgical intervention. Autologous nerve grafting remains the gold standard for treating PNI in the clinic, however, with many drawbacks. The construction of microenvironment to support nerve regeneration is an important strategy, in which self-assembling peptide nanofiber hydrogels are widely used. Therefore, we fabricated a self-assembling peptide hydrogel dual-functionalized with IKV and RGI in chitosan tubes for peripheral nerve repair. Results suggested that the nanofiber hydrogels could obviously promote the myelination of Schwann cells *in vitro* and facilitate the neurite growth and functional recovery* in vivo*.

In this study, the nanostructure and mechanical properties of self-assembling peptide hydrogels based on RAD have been investigated in detail. The hydrogels exhibited porous structure similar to ECM, with pores ranging from 10 nm to 200 nm, in which those ranging from 25 nm to 100 nm accounted for a large proportion (**Figure S9**). There was no significant difference in pore size among all groups. The sizes of the fibers and pores of a porous scaffold are quite important for cell growth and migration. Zhang et al. discussed cell migration behavior within the self-assembling peptide nanofiber hydrogels previously 67. Cells attached on microfibers that are similar in size to most cells (~5 - 30 μm) are in fact in a 2D environment with a curvature depending on the diameter of the microfibers. For a true 3D environment for cell growth and good nutrition diffusion, a scaffold's fibers and pores must be much smaller than the cells, so that the cells are surrounded by the scaffold, similar to the native extracellular matrix. Cells can only attach to the microfibers, while they can be fully embedded in the nanofiber scaffolds, where they can still move freely without hindrance. The self-assembling peptide hydrogels consisting of interwoven nanofibers ~ 10 nm in diameter with pore size ~ 10 - 200 nm could provide a true 3D environment for cell growth. And our previous work demonstrated that functionalized self-assembling peptide hydrogels could promote cell attachment, proliferation and migration of multiple cell types including osteoblasts 68, human umbilical vein endothelial cells 69, and human adipose stem cell 50. These cells seeded on the surface of the hydrogels could migrate deep into the hydrogels and the cells embedded within the hydrogels could also migrate and form cell connections. Besides, Schwann cells could spread inside the hydrogel and the DRG neurites also could elongated on the hydrogel *in vitro* 37. Additionally, the degradation of hydrogels is also very important for nerve regeneration* in vivo*. Our *in vitro* experiment indicated no obvious degradation could be observed in PBS at 37 °C for 14 days (data not shown). *In vivo*, the environment of SAP hydrogel is much more complex than that *in vitro* so that the hydrogel showed good degradation 70. After the implantation of nerve conduits with SAP hydrogels, Schwann cell, endothelial cells and fibroblasts migrate fast into the conduits and secret various enzymes. The changing pH, ionic strength, and the enzymes make hydrogels undergo gradually degradation and then contribute to provide an appropriate microenvironment for axon regeneration. Therefore, the hydrogels could provide a suitable 3D microenvironment similar to native ECM for cell migration and would not hinder the growth of neurites in the process of nerve regeneration.

The biophysical properties of biomaterials are crucial design criteria in the preparation of artificial regenerative niches for nerve regeneration. Neural regenerative biomaterials should have an elasticity that mimics the soft extracellular matrix of neural tissue, which has an elasticity of 0.5~3 kPa 71. The moduli of hydrogels in this study had similar viscoelastic values as neural tissue, contributing to nerve growth (**Figure S10**). Results also showed that the G' of RAD/IKV hydrogel was the highest among all the hydrogels. To our knowledge, the electrostatic repulsive force confronts the hydrophobic attraction to form fine nanofibers in the hydrogels, and the mechanical properties of the hydrogel are affected by electrostatic interaction and hydrophobic interaction between peptides 23, 72. The net charge and hydrophobicity of the peptides in this study are listed in **Table S2**. The IKVAV motif has a high proportion of nonpolar amino acids such as Val, Ala, and Ile, which results in strong hydrophobic forces. In RAD/IKV hydrogel, the hydrophobic attraction can enhance the assembly and aggregation of nanofibers, causing high G' value. However, the RGIDKRHWNSQ and IKVAV-GG-RGIDKRHWNSQ motifs have high net charge and high hydrophilic residue ratio, which contributes to enhance electrostatic repulsive force and hinder the aggregation of nanofibers in RAD/RGI, RAD/IKV-GG-RGI, and RAD/IKV/RGI hydrogels. Additionally, the Circular Dichroism result showed that the β-sheet content of RAD/IKV was the highest among all groups, which contributed to the high G', as shown in **Table S3**.

The Schwann cells are able to wrap around the axons of neurons to form compact myelin sheaths, allowing for rapid and salutatory conduction of electrical impulses and support the integrity of axons in the PNS. In these process, Schwann cells go through changes in morphology and gene expression 55. We focused on the effects of dual-functionalized SAP hydrogels on the maturation, myelination, and neurotrophin secretion of SCs *in vitro*. After a 7-day incubation, SCs in different groups displayed different morphologies. On hydrogels with both IKV and RGI, improved cell adhesion was observed accompanied by increased cell spreading and elongation relative to other groups, which was consistent with the morphology of mature SCs involved in myelination 58. Moreover, RSCs cultured on hydrogels with both IKV and RGI showed enhanced gene expression of NGF, BDNF, CNTF, PMP22, and NRP2 as compared with those cultured on other three groups. Additionally, the expression of NCAM, a marker gene only expressed in immature RSCs, was decreased by > 0.25-fold on dual-functionalized hydrogels relative to its expression in RSCs cultured on other hydrogels. The secretion of related neurotrophins including NGF, BDNF, and CNTF showed the consistent results with gene expression, with RAD/IKV-GG-RGI and RAD/IKV/RGI groups the highest. These results indicated that IKV and RGI had a positive synergetic effect on RSCs myelination and neurotrophin secretion.

Multiple genes are upregulated or downregulated following PNI, especially in 14 days postoperatively, and reflected the *in vivo* regenerative process and mechanism 73-75. Previous studies reported a maximum number of differentially expressed genes at 7-days post-injury. Therefore, we harvested the nerve grafts from each group and evaluated gene expression related to growth factors (NGF, BDNF, and VEGF) and myelination (PMP22, P0, and S100) inside the conduits at 7-days post-operation (**Figure 5**). Studies showed that NGF and BDNF are very important for the development and regeneration of the nervous system, and they were kept up-regulated after sciatic nerve injury 76. The expression of growth factors and myelin-related genes were significantly higher in the Autograft group relative to all other groups. Specifically, we found that VEGF expression was similar among the Autograft group and functionalized peptide groups, indicating an improvement in vascularization that could contribute to nerve regeneration. The expression of S100 in groups containing both IKV and RGI was significantly higher than that in groups containing either IKV or RGI hydrogels, suggesting enhanced SC proliferation *in vivo*. There was no significant difference among functionalized hydrogels in the expression of neurotrophins and myelination genes, but obvious higher means of BDNF expression in RAD/IKV-GG-RGI and RAD/IKV/RGI groups could be seen. The results confirmed the outstanding outcome of autograft in repairing nerve injury. And a trend to approach the Autograft group was obvious in dual-functionalized peptide groups. This may could explain the difference in regeneration between Autograft group and dual-functionalized peptide groups after 12 weeks. Additionally, the cell types and the proportion of each cell type in the lesion side are very important to evaluate the gene expression, such as Schwann cells, macrophage, and neurons 77. The secretion of NGF and BDNF, and the myelination were considered as the function of SCs. Therefore, here we only discussed the effect of hydrogels on SCs after 7 days, which was similar to previous study 78. However, more investigation about the relationship between cell types and gene expression* in vivo* should be carried out.

To evaluate the functional recovery of each group, we measured the morphometric parameters of the regenerated nerves (**Figure 6**), their electrophysiological performance (**Figure 7**), the innervated muscle weight and remodeling of muscle fibers, and motor function (**Figure 8**). The results indicated that the functional recovery in dual-functionalized groups was significantly improved compared with those in the Hollow, RAD/IKV, and RAD/RGI groups, approaching that of the Autograft group. Axonal area and myelin sheath thickness are direct indicators of nerve conduction velocity maturity according to studies 79. Regenerating axons have smaller diameters and higher resulting axoplasmic resistances, while fully matured axons have wider diameter and lower subsequent resistance 80. Myelin thickness is proportional to the axonal diameter and the intermodal length, and the ratio is similar in general, which is about 0.6 64. Although the nerve fiber diameter and the myelin sheath thickness in RAD/IKV/RGI group were significantly lower than the Autograft group, there was no significantly difference in g-ratios among dual-functionalized groups and Autograft group, indicating the consistent myelination degree in these groups. Notably, the g-ratios of Hollow group were also comparable to that of the Autograft group. One possible explanation for this finding could be the significantly lower nerve fiber diameter and myelin sheath thickness in the Hollow group, suggesting the regenerated nerve in the Hollow group had reached a mature state and could not get bigger. At the same time, the nerve fiber density in the Hollow group was significantly lower than other groups, suggesting the limited regeneration of new nerves. Additionally, the g-ratios of RAD/IKV and RAD/RGI groups were significantly higher than those of RAD/IKV-GG-RGI and Autograft groups, indicating the less mature myelination, but the nerve fiber diameter of the two groups was significantly higher than that of Hollow group, thus we think the nerves of the single functionalized groups may keep growing with time. The re-innervation of the muscle distal to the injured nerve was assessed with CMAP recordings. In consistent with the myelination degree, the CMAP latency ratios of the dual-functionalized groups were comparable to that of Autograft group. The CMAP latency ratio of the Hollow group was significantly higher than the RAD/IKV-GG-RGI, RAD/IKV/RGI, and Autograft groups, possibly caused by the tiny nerve fiber, low regenerated nerve density or mismatch of the regenerated nerves and muscle. The amplitude of the CMAP could reflect the motor unit numbers that regenerated 80. There was no significant difference between dual-functionalized groups and Autograft group in the CMAP amplitude ratio, indicating that the numbers of motor units regenerated for these groups were similar. The CMAP amplitude ratios in the Hollow, RAD/IKV, and RAD/RGI groups were significantly lower than the other groups, suggesting less motor regenerated nerves. The recovery of motor function was evaluated via muscle histology and the motion of rats. After nerve injury, target muscle loses innervation of the nerve and it will get re-innervation when the regenerating axon grows and is accepted; at the same time, target muscle gets atrophied. The atrophied target muscle can prevent re-innervation, and if re-innervation is delayed, the axons are unable to recover the function completely 81. Therefore, the speed of nerve regeneration is very important, and the muscles and the regenerated nerves are closely related in achieving nerve regeneration and function recovery. The average cross-sectional area of the dual-functionalized groups was not significantly different from the Autograft group, while the wet weight ratio was significantly lower, indicating that the muscle in the dual-functionalized groups went through atrophy and lost some muscle fibers. However, the muscle in the Hollow, RAD/IKV, and RAD/RGI groups went through more severe atrophy than the other groups. The motor recovery evaluated by SFI was consistent with the muscle recovery. The combined results from different assessments provided supporting evidence for the efficacy of the dual-functionalized peptide hydrogels with IKVAV and RGI to bridge sciatic nerve gaps successfully and efficiently. However, we have done *in vivo* study for RAD hydrogel and the results were not comparable to functionalized hydrogels, with a regenerative effect similar to hollow chitosan tube. And the purpose of our study was to investigate the synergetic effect of IKVAV and RGI, which have been confirmed previously compared with pure RAD 23, 36. The RAD/IKV and RAD/RGI groups could serve as the control groups. Therefore, the RAD group was not necessary and we did not include it in this study.

To confirm the synergistic effect of IKVAV and RGI, we synthesized the dual-functionalized peptide RAD-IKV-GG-RGI as a control group, in which the IKVAV and RGI motifs were combined covalently to ensure the same quantity of the two motifs. Different from RAD/IKV-GG-RGI hydrogel, RAD-IKV, RAD-RGI and RAD co-assembled to form a homogeneous RAD/IKV/RGI hydrogel with more β-sheet content (45.9%) than the RAD/IKV-GG-RGI hydrogel (40.3%, **Table S3**). The cell elongation length of RSCs on the RAD/IKV/RGI hydrogel was significantly longer than that on the RAD/IKV-GG-RGI hydrogel (**Figure 2C**), although PMP22, NRP2, and BDNF expression in RSCs cultured on the RAD/IKV-GG-RGI hydrogel was significantly higher than that on the RAD/IKV/RGI hydrogel (**Figure 3C, D, G**). And the neurotrophin secretion of NGF, BDNF, and CNTF was not significantly different between the two groups, respectively (**Figure 4**). In the first week after surgery, the gene expression of the RAD/IKV/RGI and RAD/IKV-GG-RGI groups was similar without significant difference (**Figure 5**). After 12 weeks, there was no significant difference in the axon re-myelination, electrophysiological performance, and functional recovery between the two dual-functionalized peptide groups (**Figure 6-8**). These results suggested that the combination methods had no effect on the synergistic function of IKVAV and RGI *in vitro* and *in vivo*. Notably, the contents of exact functionalized motifs were different between RAD/IKV-GG-RGI and RAD/IKV/RGI because RGI has higher molecular weight than IKVAV. Generally, there were more RGI motif and less IKVAV motif in RAD/IKV-GG-RGI hydrogel than those in RAD/IKV/RGI hydrogel, which might explain the differences in gene expression. However, longer peptide chains increase the difficulty of synthesis and purification, and block the self-assembly of peptides. In consideration of the similar results in RAD/IKV/RGI and RAD/IKV-GG-RGI groups, we think the co-assembling of RAD-IKV, RAD-RGI, and RAD was a more beneficial way to construct dual-functionalized peptide hydrogel.

Combined use of the LN or LN peptide fragment (IKVAV) and BDNF for nerve repair has been previously investigated. Park et al. fabricated a hyaluronic acid-based hydrogel with IKVAV peptide and BDNF for use as a 3D biomimetic scaffold for the treatment of spinal cord injury, resulting in foster differentiation of stem cells and facilitated nerve regeneration 82. Frick et al. developed an injectable IKVAV-functionalized self-assembling peptide hydrogel incorporated with BDNF that promoted attachment and neurite outgrowth of spiral ganglion neurons for the treatment of cochlear implants 83. Moreover, LN-binding BDNF constructed via fusing with laminin-binding domain (LBD) to BDNF (LBD-BDNF) was widely applied for the treatment of cerebral ischemia and reperfusion injury, facial nerve injury, and recurrent laryngeal nerve injury 33, 84-86. They showed that LBD-BDNF was able to target accumulated LN to exert targeted therapy of injured neurons. It is possible that combined use of LBD-BDNF and LBD-CNTF might promote the ordered growth of axons and greatly enhance facial nerve regeneration. However, there are numerous problems associated with clinical applications of exogenous growth factors. Most growth factors degrade rapidly *in vivo* due to their short half-life and fast diffusion, and the use of high doses or periodic injections is expensive and impractical. Unlike the soluble growth factors, bioactive motifs are widely used as the alternative, which could be covalently conjugated on biomaterials 87. It's proven that the conjugated bioactive peptides exerted* in situ* and preserved their activities in a long period. The bioactive peptide motif was covalently linked to the C-terminal of the self-assembling peptide which could form well-defined nanofibrous hydrogels capable of providing a 3D microenvironment similar to native ECM. Previous study showed that the release of bioactive peptide motif in self-assembling peptide occurred along with the degeneration of the hydrogel 62. Additionally, the bioactive peptide motifs could function before the degradation of the hydrogel. Therefore, using the BDNF mimicking epitope RGI combined with IKVAV, we not only maintained the synergetic effect of the two components, simplified the experimental process, and reduced BDNF-related side effects but also fabricated hydrogels with 3D structure necessary for cell growth.

From nerve regeneration especially PNS regeneration strategies, the design of biological materials requires full consideration of regenerative microenvironment, such as simulation of extracellular matrix by modifying scaffolds with IKVAV and RGD, addition of growth factors or peptide motifs derived from growth factors for the insufficient neurotrophic factor in this study, modification with antibodies to the nogo66 receptor to shield suppressor for the suppression of microenvironment, and presenting aligned topography and soft stiffness in our previous study 11, 88. In a word, the nerve regeneration is very complex, and the function of single factor is not enough. The synergy of various factors should be considered carefully. Therefore, it's of vital importance to know if and how these two or more regulatory signals have co-effects on tissue regeneration, which will provide promising strategies to design ideal biomaterials by mimicking native regenerative microenvironment. To our knowledge, nerve regeneration will either not occur or be dysfunctional without either neurite outgrowth matrix or neurotrophic factors. This suggests a synergistic effect on nerve regeneration between growth-promoting matrix and neurotrophin. Additionally, Schwann cells play important roles in the secretion of growth-promoting matrix, neurotrophic factors, and cell adhesion molecules. Therefore, the main aim of this study was to construct the microenvironment suitable for Schwann cell growth, which could simulate the regeneration process *in vivo*. The self-assembling peptide dual-functionalized with IKVAV and RGI could enhance the adhesion, maturation and neurotrophin secretion of Schwann cells, which was beneficial for neurite growth. Additionally, the adhesion and extension of neurite is important for the bridge of new nerve. IKVAV motif could provide the adhesion domain for Schwann cells and neurite while RGI motif could promote the neurite growth with the similar effect as BDNF. The synergistic effect of two functionalized self-assembling peptide was not first proposed in this study. Sun et al. developed SAP hydrogels with RGD and IKVAV motifs for nerve regeneration, which could enhance SC recruitment 23. Lu et al. applied RGI and VEGF-mimetic peptide (KLT) epitopes to modify RAD peptide to construct neurovascular microenvironment for nerve repair, which could regulate and direct neural and vascular cell behavior and improve the recovery effect synergistically 13. Lu et al. combined RGI and NGF-mimetic peptide (CTD) to synergistically improve the regeneration of sensory and motor nerves 37. IKVAV and RGD are mainly short peptides related to extracellular matrix, which promote nerve regeneration by enhancing their interaction with cells, while RGI, KLT, and CTD are mainly growth factor mimicking peptides, which simulate growth factors to promote nerve regeneration and vascularization. The regenerative strategy was different from the study which used the KLT and RGI motifs for constructing the neurovascular microenvironment, for we didn't involve the factor for vascular regeneration. Compared with the study by Sun et al., the functional motifs they used were RGD and IKVAV from extracellular matrix, which both promote the adhesion of cells. However, the functional motifs in our study were from laminin and BDNF, belonging to the extracellular matrix protein and neurotrophin, respectively, which were from different cues and had a synergistic effect.

In summary, our findings demonstrated the efficacy of the dual-functionalized SAP scaffolds combining IKVAV and RGI to support myelination and neurotrophin secretion *in vitro* and *in vivo* and promote functional recovery of sciatic nerve injury, which could be a promising and effective strategy for nerve regeneration. However, we were unable to elucidate the precise regeneration mechanisms associated with this the synergetic effect in the article, and there remain other potential improvements we can make, such as the addition of angiogenic factors, appropriate cellular support, and aligned nanofiber. Therefore, further studies are required.

## Conclusions

In this study, we fabricated dual-functionalized self-assembling peptide nanofiber hydrogels by mixing RAD with the LN-derived peptide epitope IKVAV and a BDNF-mimetic peptide epitope RGI, respectively. We found that the dual-functionalized SAP hydrogels promoted RSC adhesion, myelination, and neurotrophin secretion *in vitro* and successfully bridged a 10-mm gap representing a sciatic nerve defect in rats *in vivo*. During the early regeneration stage, the hydrogel enhanced myelination-related and neurotrophin-related gene expression *in vivo*. Histologic and morphologic analysis, as well as electrophysiological and behavioral examinations after graft implantation, indicated that chitosan tubes filled with the dual-functionalized hydrogels displayed good recovery outcomes. Therefore, our study demonstrated the synergistic effect of IKVAV and RGI on axonal regrowth and function recovery after PNI, which will have potential applications in nerve tissue engineering.

## Supplementary Material

Supplementary methods, figures and tables.

## Figures and Tables

**Table 1:** Self-assembling peptides used in this study

Name	Sequences	Description
RAD*	Ac-(RADA)_4_-NH_2_	Self-assembling backbone
RAD-IKV*	Ac-(RADA)_4_-G_2_-IKVAV-NH_2_	Laminin mimicking peptide
RAD-RGI*	Ac-(RADA)_4_-G_2_-RGIDKRHWNSQ-NH_2_	BDNF mimicking peptide
RAD-IKV-GG-RGI*	Ac-(RADA)_4_-G_2_-IKVAV-G_2_-RGIDKRHWNSQ-NH_2_	Dual-functionalized peptide
RAD/IKV	-	50% RAD + 50% RAD-IKV
RAD/RGI	-	50% RAD + 50% RAD-RGI
RAD/IKV-GG-RGI	-	50% RAD + 50% RAD-IKV-GG-RGI
RAD/IKV/RGI	-	50% RAD + 25% RAD-IKV + 25% RAD-RGI

*The concentration of the peptide solution is 1% (w/v).

## Abbreviations

3D: three-dimensional
AFM: atomic force microscopy
ANOVA: analysis of variance
BDNF: brain-derived neurotrophic factor
BSA: bovine serum albumin
CD: circular dichroism
CMAPs: compound muscle action potentials
CNS: central nervous system
CNTF: ciliary neurotrophic factor
DMEM: Dulbecco's Modified Eagle Medium
ECM: extracellular matrix
ELISA: enzyme-linked immunosorbent assay
ESI-MS: electrospray ionization mass spectrometry
FBS: fetal bovine serum
FN: fibronectin
HPLC: high-performance liquid chromatography
IGF-2: insulin-like growth factor-2
IKV: IKVAV
LBD: laminin-binding domain
LH: left hind paw
LN: laminin
MBP: myelin basic protein
NCAM: neuronal cellular adhesion molecules
NGCs: nerve guidance conduits
NGF: nerve growth factor
NRP2: neuropilin 2
PA: peptide amphiphile
PBS: phosphate buffered saline
PMP22: peripheral myelin protein
PNI: peripheral nerve injury
PNS: peripheral nervous system
PS: Penicillin-Streptomycin
PTEF: polytetrafluoroethylene
qRT-PCR: quantitative real-time polymerase chain reaction
RAD: Ac-(RADA)_4_-NH_2_
RGI: RGIDKRHWNSQ
RH: right hind paw
RSCs: rat Schwann cells
SAP: self-assembling peptide
SCs: Schwann cells
SD: standard deviation
SEM: scanning electron microscopy
SFI: sciatic function index
TEM: transmission electron microscope
VEGF: vascular endothelial growth factor
