# Supplementary material for: Self-assembling peptide hydrogels functionalized with LN- and BDNF- mimicking epitopes synergistically enhance peripheral nerve regeneration
Source: Theranostics. 2020 Jul 9;10(18):8227–49. doi: 10.7150/thno.44276 (PMC7381722; doi:10.7150/thno.44276)
Supplement: Supplementary file 1 — Supplementary methods, figures and tables. [file thnov10p8227s1.pdf]

Supporting Information

Self-assembling peptide hydrogels functionalized with LN- and BDNF- mimicking epitopes synergistically enhance peripheral nerve regeneration

Shuhui Yang[1*], Chong Wang[2*], Jinjin Zhu[3*], Changfeng Lu[2,4], Haitao Li[2], Fuyu Chen[2], Jiaju Lu[1], Zhe Zhang[1], Xiaoqing Yan[1,5], He Zhao[1], Xiaodan Sun[1], Lingyun Zhao[1], Jing Liang[6], Yu Wang[2✉], Jiang Peng[2✉], and Xiumei Wang[1✉]

1. State Key Laboratory of New Ceramics and Fine Processing, Key Laboratory of Advanced Materials of Ministry of Education, School of Materials Science and Engineering, Tsinghua University, Beijing 100084, China
2. Institute of Orthopedics, Chinese PLA General Hospital, Beijing 100853, China; Co-innovation Center of Neuroregeneration, Nantong University, Nantong, Jiangsu Province 226007, China
3. Department of Orthopaedic Surgery, Sir Run Run Shaw Hospital, Zhejiang University School of Medicine & Key Laboratory of Musculoskeletal System Degeneration and Regeneration Translational Research of Zhejiang, Hangzhou 310016, China
4. Department of Orthopaedics and Trauma, Peking University People's Hospital, Beijing 100191, China
5. School of Clinical Medicine, Tsinghua University, Beijing 100084, China
6. Department of Pediatrics, Tianjin Hospital, Tianjin University, No. 406 Jiefang Nan Road, Tianjin 300211, China

* Contributed equally to this work

✉ Corresponding author: Xiumei Wang, E-mail: wxm@mail.tsinghua.edu.cn, Phone: 86-10-62782966. Fax: 86-10-62771160. Jiang Peng, E-mail: pengjiang301@126.com. Yu Wang, E-mail: wangwangdian628@126.com.

Methods

Cell proliferation assay

The proliferation of RSCs on different hydrogels was evaluated. 50 μL of peptide solution was loaded directly into the wells of a 96-well plate, and then 100 μL of culture medium was gently added onto the peptide solution. After incubation for 15 min at 37 °C, the medium was removed and changed at least twice to equilibrate the hydrogels. RSCs were seeded on the surface of the hydrogel at a density of 8×10^3 cells/well (n = 4), and the medium was changed with fresh medium every 2 days. Cell proliferation in different groups was assessed using Cell Counting Kit-8 (CCK-8, DOJINDO, Tokyo, Japan). After culture for 1, 4, and 7 days, the samples were incubated with 10% CCK-8 working solution in cell culture medium for 3 h at 37 °C. Then, 100 μL of the supernatant was transferred to a new 96-well plate, and the absorbance was measured at 450 nm using an EnSpire Multimode Plate Reader (PerkinElmer, USA).

Table S1. Primer sequences used for quantitative RT-PCR

Gene	Primers (F=forward, R=reverse)
GAPDH	F: ATGATTCTACCCACGGCAAG
	R: CTGGAAGATGGTGATGGGTT
NGF	F: CGCTCTCCTTCACAGAGTTTT
	R: GACATTACGCTATGCACCTCAGA
BDNF	F: TCTACGAGACCAAGTGTAATCCCA
	R: CTTATGAACCGCCAGCCAAT
CNTF	F: ATGGCTTTCGCAGAGCAAAC
	R: CAACGATCAGTGCTTGCCAC
IGF-2	F: GAACAACAATAGCCGCCCAAACTC
	R: CATGTTCTGTTCCTCTCCTTGGGT
S100	F: GTTGCCCTCATTGATGTCT
	R: CTGCTCTTTGATTTCCTCC
MBP	F: TCTGGCAAGGACTCACACAC
	R: AAATCTGCTGAGGGACAGGC
NCAM	F: TTCAGTGACGACAGTTCGGAGC
	R: TGCGAAGACCTTGAGGTGGAT
PMP22	F: TGTACCACATCCGCCTTGG
	R: GAGCTGGCAGAAGAACAGGAAC
NRP2	F: CTTGCTCCCTCTTTGCTG
	R: TTCCTTGTGGTGTCTTCTG
VEGF	F: ACCATGCCAAGTGGTGAAGT
	R: GGGCTTCATCATTGCAGCAG
P0	F: AAGTCTATGGTGCTGTGGGC
	R: CCCATACCTAGTGGGCACTTT

Table S2. Physicochemical properties of self-assembling backbone and functional motifs

Sequences	Net charge at pH=7	Hydrophilic residue ratio	Description
$(RADA)_4$	0.0	50%	Backbone
G_2-IKVAV	1.0	14%	LA motif
G_2-RGIDKRHWNSQ	2.1	54%	BDNF motif
G_2-IKVAV-G_2-RGIDKRHWNSQ	3.1	40%	Dual-motif

Table S3. Estimated secondary structure fractions of different peptides

Peptides	Secondary structure fractions (%)*					
	H (r)	H (d)	S (r)	S (d)	Turn	Unrd
RAD	4.8	0.0	32.3	14.1	15.4	33.4
RAD-IKV	8.5	11.4	0.0	8.4	24.9	46.9
RAD-RGI	4.8	1.9	0.0	6.1	32.2	55.0
RAD-IKV-GG-RGI	3.7	6.3	11.4	9.2	26.4	43.1
RAD/IKV	6.5	0.0	36.4	15.8	12.3	29.0
RAD/RGI	1.9	2.7	33.8	14.1	15.9	31.7
RAD/IKV-GG-RGI	2.5	0.0	28.6	11.7	20.4	36.9
RAD/IKV/RGI	2.1	0.1	32.8	13.1	19.4	32.4

*Abbreviations: H (r), regular α-helix; H (d), distorted α-helix; S (r), regular β-strand; S (d), distorted β-strand; Turn, β-turn structure; Unrd, unordered structure. Total Sheet content = S (r) + S (d).

Table S4. Nanofiber width from molecular models and AFM measurements

Peptides	W_{the}*	W_{adj}**	W_{mea}
RAD	5.9 nm	12.1 nm	15.4 ± 2.1 nm
RAD-IKV	8.3 nm	14.5 nm	-***
RAD-RGI	10.5 nm	16.7 nm	-
RAD-IKV-GG-RGI	13.0 nm	19.2 nm	-
RAD/IKV	10.7 nm	16.9 nm	18.1 ± 1.9 nm
RAD/RGI	15.1 nm	21.3 nm	23.0 ± 1.5 nm
RAD/IKV-GG-RGI	20.1 nm	26.3 nm	29.6 ± 2.2 nm
RAD/IKV/RGI	15.1 nm	21.3 nm	21.6 ± 0.9 nm

*According to the molecular model proposed in Figure 1A.

**W_{the}: theoretical fiber width; W_{mea}: measured fiber width by AFM; W_{adj}: adjusted theoretical fiber width considering the size effect of the AFM probe tip based on the estimation $W_{adj} = W_{the} + 2\ (2R_tH - H^2)^{1/2}$, R_t = AFM tip size (~ 10 nm), H = sample height (~ 0.5 nm) [1, 2].

***No nanofiber formation was observed.

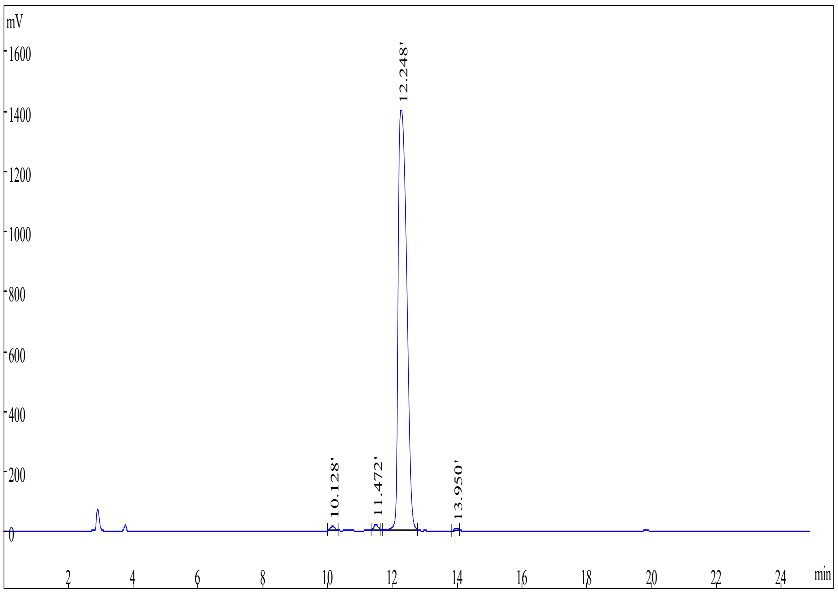

Figure S1. Analytical HPLC trace of RAD peptide.

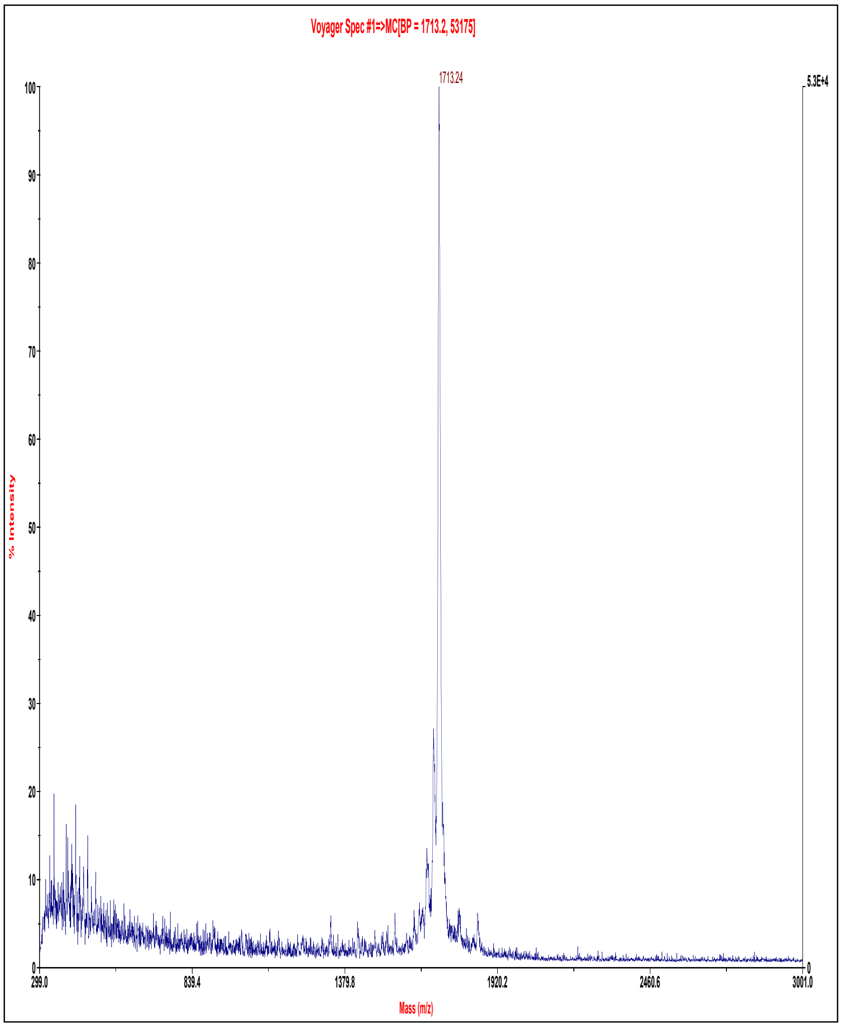

Figure S2. The electrospray ionization mass spectrometry (ESI-MS) of RAD peptide.

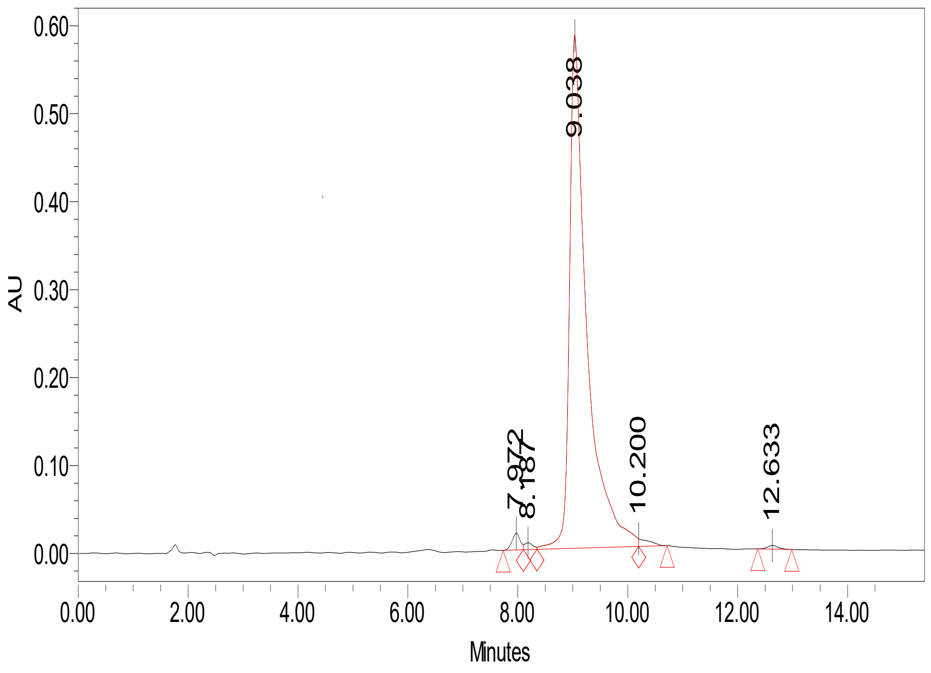

Figure S3. Analytical HPLC trace of RAD-IKV peptide.

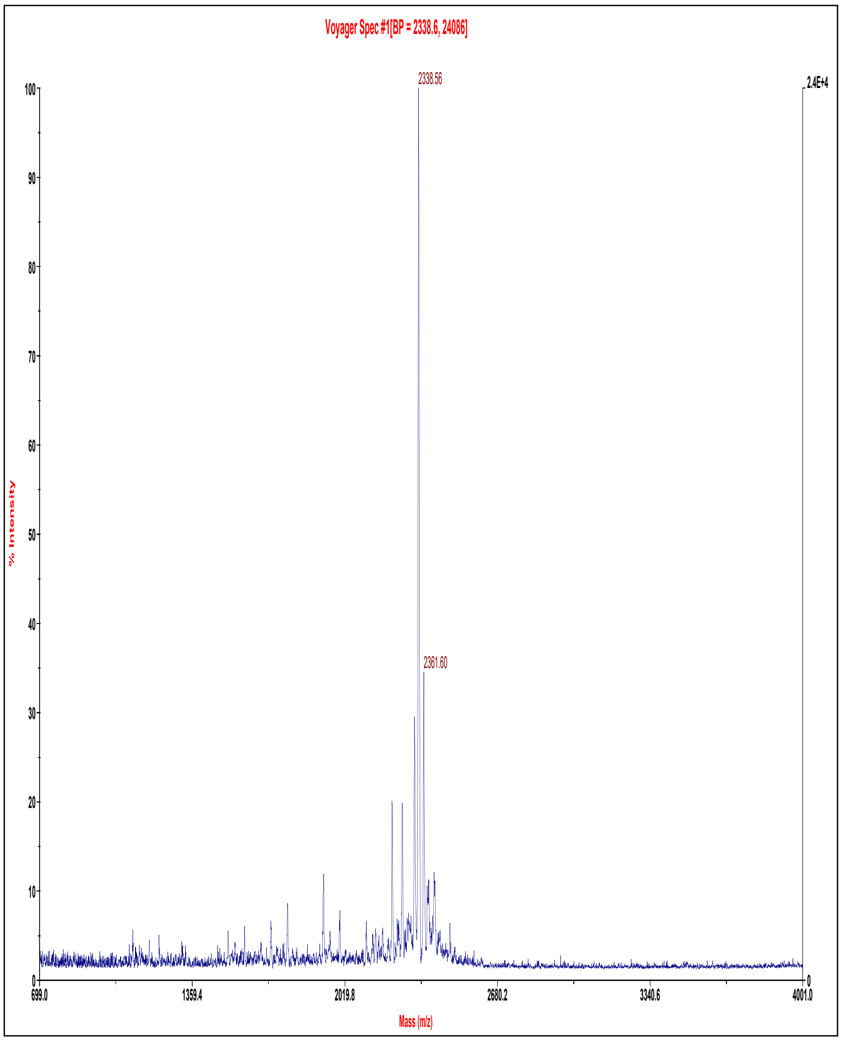

Figure S4. The electrospray ionization mass spectrometry (ESI-MS) of RAD-IKV peptide.

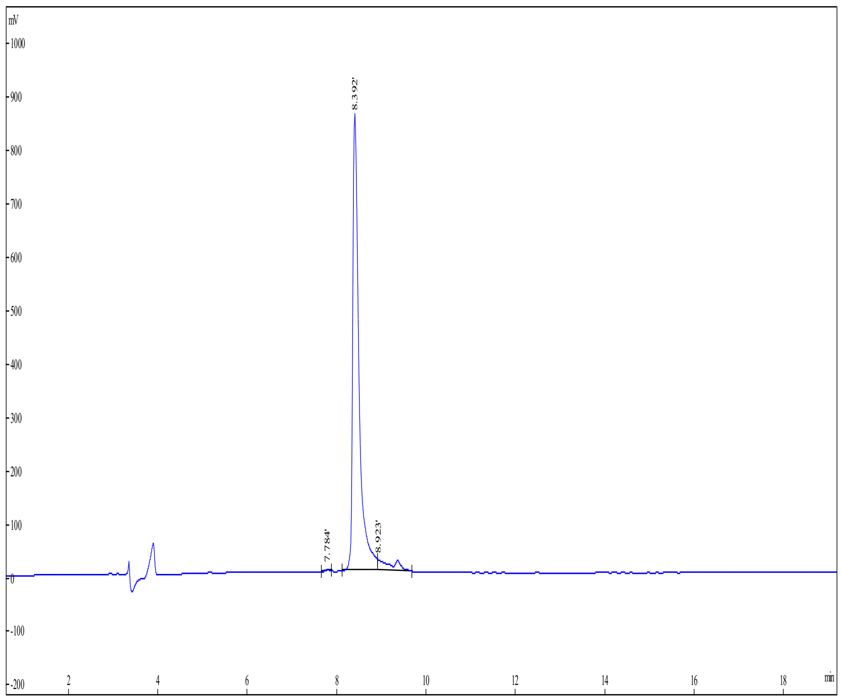

Figure S5. Analytical HPLC trace of RAD-RGI peptide.

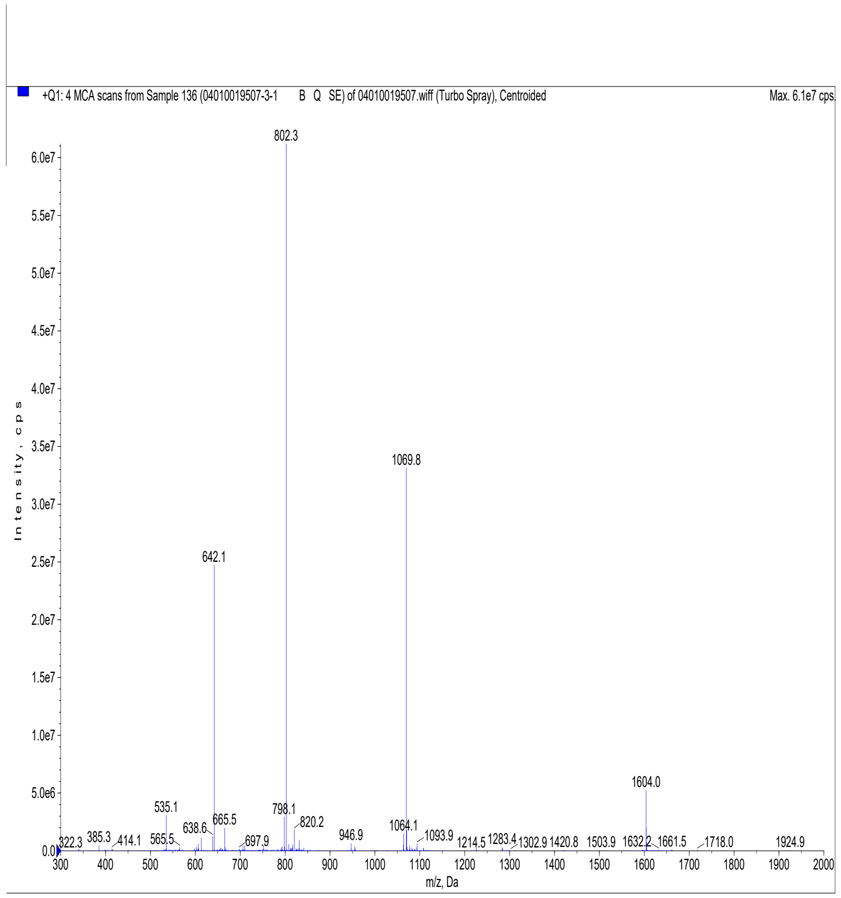

Figure S6. The electrospray ionization mass spectrometry (ESI-MS) of RAD-RGI peptide.

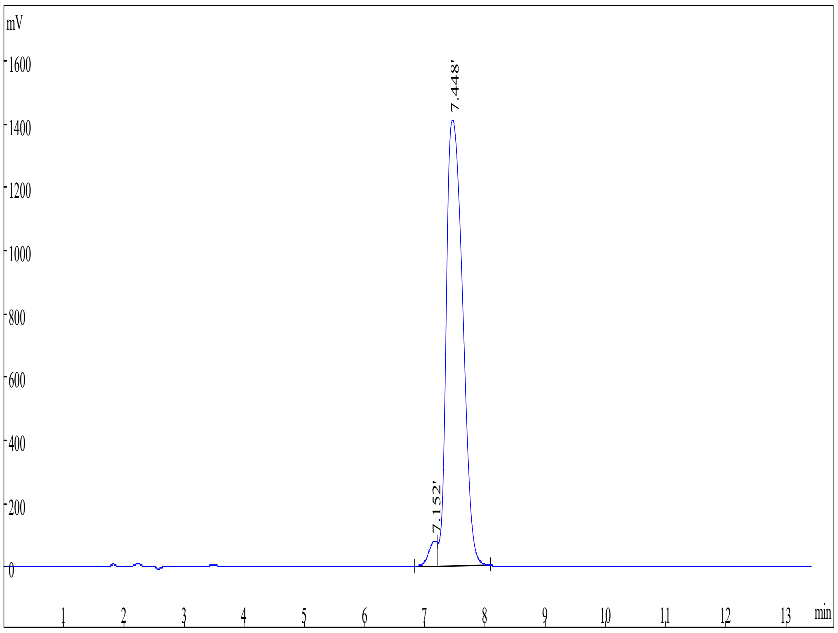

Figure S7. Analytical HPLC trace of RAD-IKV-GG-RGI peptide.

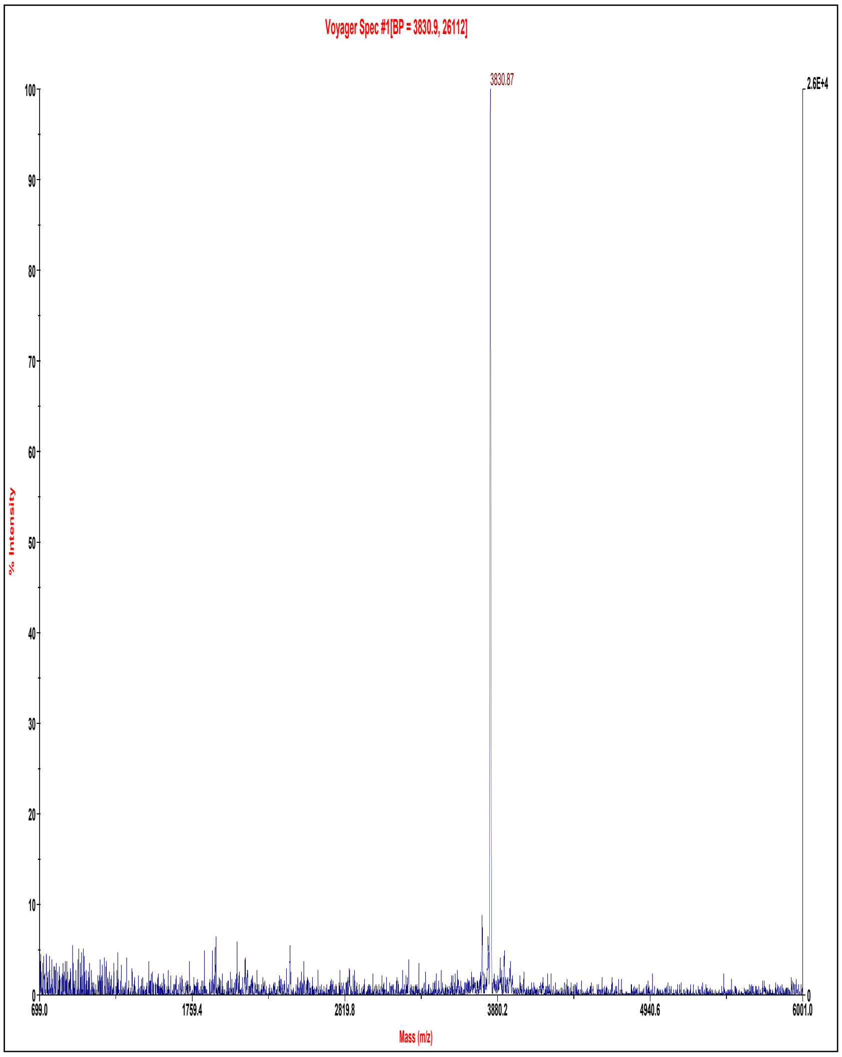

Figure S8. The electrospray ionization mass spectrometry (ESI-MS) of RAD-IKV-GG-RGI peptide.

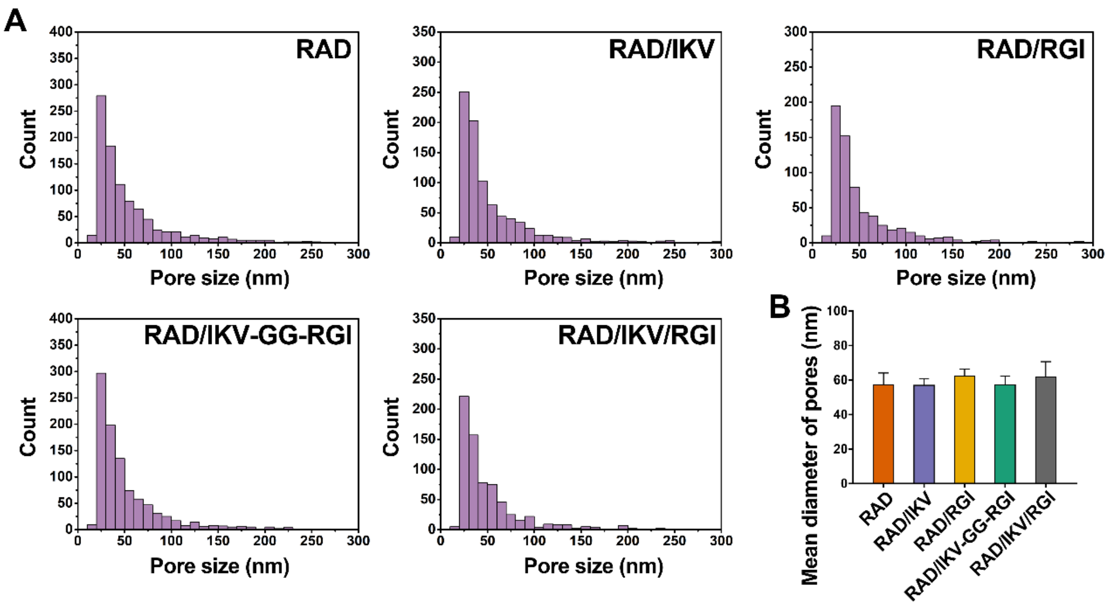

Figure S9. The size of pores in different self-assembling peptide hydrogels. (A) Typical distribution of pore sizes in different groups. (B) Analysis of the mean diameter of pores. Values are represented as mean ± SD. Statistical analysis was carried out using one-way analysis of variance (ANOVA), followed by Tukey's post hoc test (n = 3).

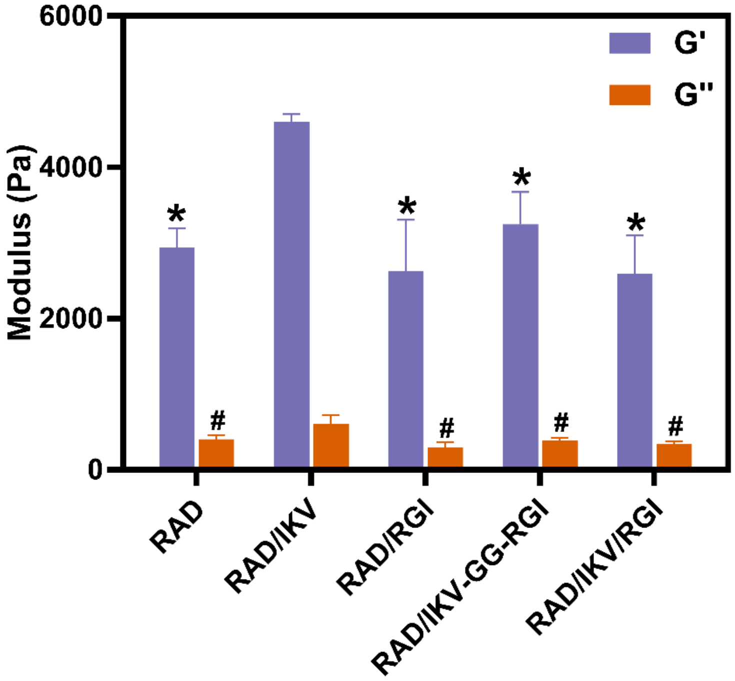

Figure S10. The average G' and G'' of self-assembling peptide hydrogels. The average G' values were respectively 2942.6 ± 250.0 Pa, 4600.0 ± 101.4 Pa, 2629.7 ± 675.1 Pa, 3246.3 ± 426.6 Pa, and 2594.8 ± 502.9 Pa. The average G'' values were respectively 406.4 ± 51.4 Pa, 606.4 ± 116.7 Pa, 298.1 ± 64.7 Pa, 386.9 ± 39.0 Pa, and 340.2 ± 35.4 Pa. Values are represented as mean ± SD. Statistical analysis was carried out using one-way analysis of variance (ANOVA), followed by Tukey's post hoc test. *$P < 0.05$, *versus* the G' of RAD/IKV hydrogel. $^{\#}P < 0.05$, *versus* the G'' of RAD/IKV hydrogel (n = 3).

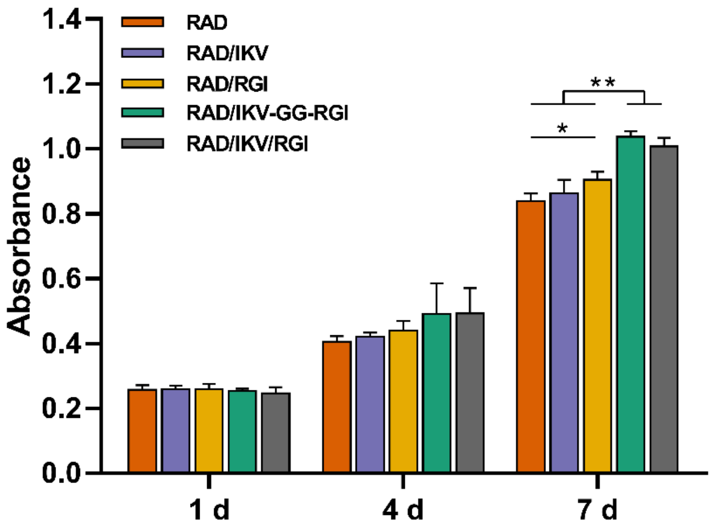

Figure S11. The proliferation of RSCs on different peptide hydrogels after 1, 4, and 7 days. Values are represented as mean ± SD. Statistical analysis was carried out using one-way analysis of variance (ANOVA), followed by Tukey's post hoc test (equal variances) or Dunnett's T3 post hoc test (unequal variances). $*P < 0.05$, and $**P < 0.01$ (n = 4).

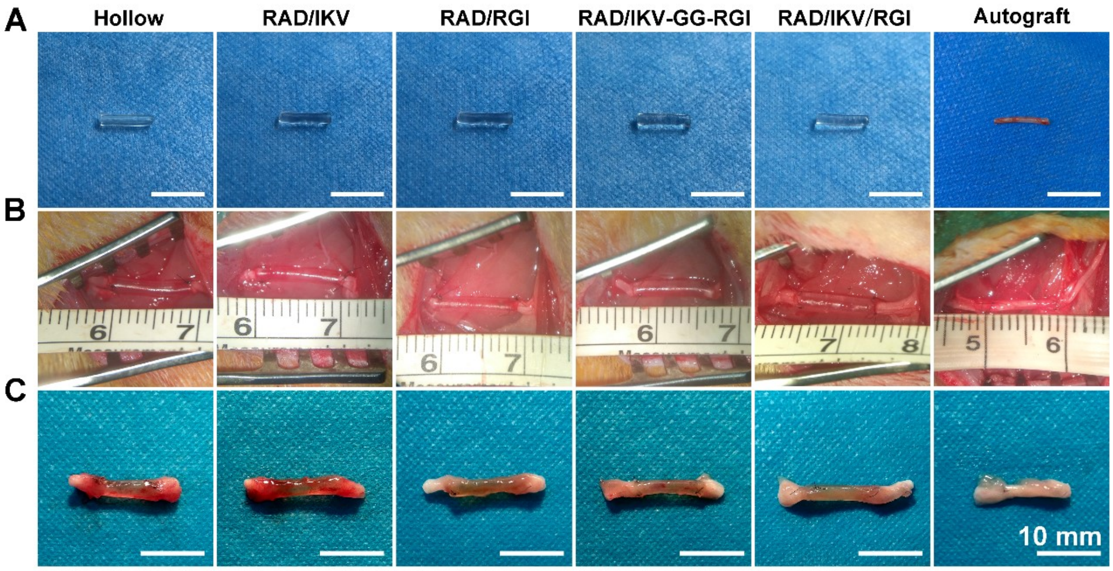

Figure S12. Nerve graft preparation (A), implantation (B) and harvest of the nerve grafts at 7 days postoperatively (C) of the different groups.

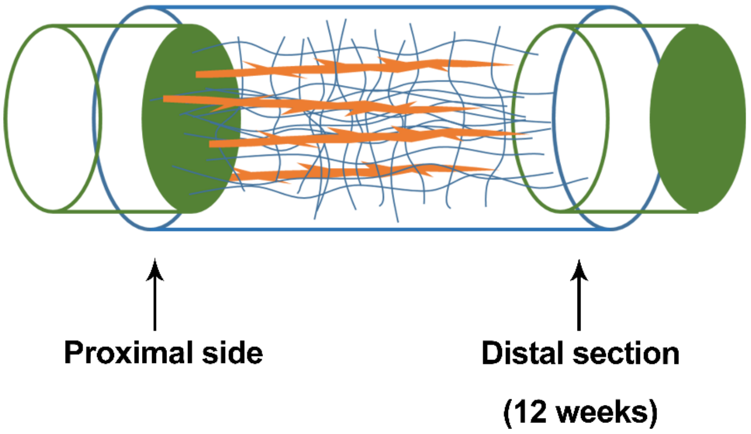

Figure S13. Scheme of transverse section of regenerated nerve at 12 weeks after surgery.

References

[1] Hong Y, Legge RL, Zhang S, Chen P. Effect of amino acid sequence and pH on nanofiber formation of self-assembling peptides EAK16-II and EAK16-IV. Biomacromolecules. 2003; 4: 1433-42.

[2] Horii A, Wang X, Gelain F, Zhang S. Biological designer self-assembling peptide nanofiber scaffolds significantly enhance osteoblast proliferation, differentiation and 3-D migration. PLoS One. 2007; 2: e190.
